# Cross-municipality migration and spread of tuberculosis in South Africa

**DOI:** 10.1038/s41598-023-29804-5

**Published:** 2023-02-15

**Authors:** Abdou M. Fofana, Harry Moultrie, Lesley Scott, Karen R. Jacobson, Anne N. Shapiro, Graeme Dor, Beth Crankshaw, Pedro Da Silva, Helen E. Jenkins, Jacob Bor, Wendy S. Stevens

**Affiliations:** 1 Wits Diagnostic Innovation Hub, Faculty of Health Sciences, University of the Witwatersrand, Johannesburg, South Africa; 2 National Health Laboratory Service, Johannesburg, South Africa; 3 Centre for Tuberculosis, National Institute for Communicable Diseases, a division of the National Health Laboratory Services, Johannesburg, South Africa; 4 Health Economics and Epidemiology Research Office, Department of Internal Medicine, School of Clinical Medicine, Faculty of Health Sciences, University of the Witwatersrand, Johannesburg, South Africa; 5 Institute for Health System Innovation & Policy, Boston University, Questrom School of Business, Boston, USA; 6 Boston University School of Public Health, Boston, USA; 7 Section of Infectious Diseases, Boston University School of Medicine and Boston Medical Center, Boston, USA

**Keywords:** Tuberculosis, Epidemiology, Epidemiology

## Abstract

Human migration facilitates the spread of infectious disease. However, little is known about the contribution of migration to the spread of tuberculosis in South Africa. We analyzed longitudinal data on all tuberculosis test results recorded by South Africa’s National Health Laboratory Service (NHLS), January 2011–July 2017, alongside municipality-level migration flows estimated from the 2016 South African Community Survey. We first assessed migration patterns in people with laboratory-diagnosed tuberculosis and analyzed demographic predictors. We then quantified the impact of cross-municipality migration on tuberculosis incidence in municipality-level regression models. The NHLS database included 921,888 patients with multiple clinic visits with TB tests. Of these, 147,513 (16%) had tests in different municipalities. The median (IQR) distance travelled was 304 (163 to 536) km. Migration was most common at ages 20–39 years and rates were similar for men and women. In municipality-level regression models, each 1% increase in migration-adjusted tuberculosis prevalence was associated with a 0.47% (95% CI: 0.03% to 0.90%) increase in the incidence of drug-susceptible tuberculosis two years later, even after controlling for baseline prevalence. Similar results were found for rifampicin-resistant tuberculosis. Accounting for migration improved our ability to predict future incidence of tuberculosis.

## Introduction

Tuberculosis (TB) prevalence varies geographically in South Africa, but the drivers of this spatial heterogeneity are poorly understood^1,2^. Like many infectious diseases, internal migration of infected people may contribute to the spread of TB and emergence of new hotspots via TB introduction and increased encounters between infectious and susceptible individuals^3–7^. In low-TB-burden countries, migrants from high-burden countries represent a large share of new infections and a focus population for treatment and preventive interventions^8–11^. As such, understanding internal migration patterns in people with TB in high-burden settings is key to preventing TB and drug-resistant TB spread^12,13^.

Previous studies have used census data, Global Positioning System (GPS) data, mobile phone call records and public transit passenger data to demonstrate increased mobility being associated with increased risk of infectious disease spread^14–20^. Existing evidence on migration and TB spread comes from genetic studies. In a study of 344 people with extensively drug-resistant (XDR) TB in KwaZulu-Natal province, South Africa, Nelson and colleagues^21^ found that 84% of genetically linked TB transmissions occurred between people living in different districts, suggesting an important role of cross-district migration in XDR-TB transmission.

Here, we aimed to (1) characterize cross-municipality migration patterns in people with laboratory-diagnosed TB, and (2) quantify the contribution of cross-municipality migration in people with TB in the spread of drug-susceptible (DS-TB) and rifampicin-resistant TB (RR-TB, resistance to rifampicin) in South Africa. We analyzed TB laboratory test results from South Africa’s National Health Laboratory Service (NHLS) database, which we linked to individuals, enabling longitudinal follow-up of people with laboratory-diagnosed TB. We identified people with laboratory-diagnosed TB who had more than one clinic visit during the study period, and we assumed that people who visited clinics located in more than one municipality are migrants. Combining laboratory-diagnosed TB results by municipality from the NHLS data, the notification:prevalence ratio from the 2018 South African TB prevalence survey, and cross-municipality migration rates from the 2016 household survey, we estimated “migration-adjusted” TB prevalence and assessed the contribution of migration to the spread of DS-TB and RR-TB.

## Results

### Migration patterns in people with laboratory-diagnosed TB

**Figure 1:**
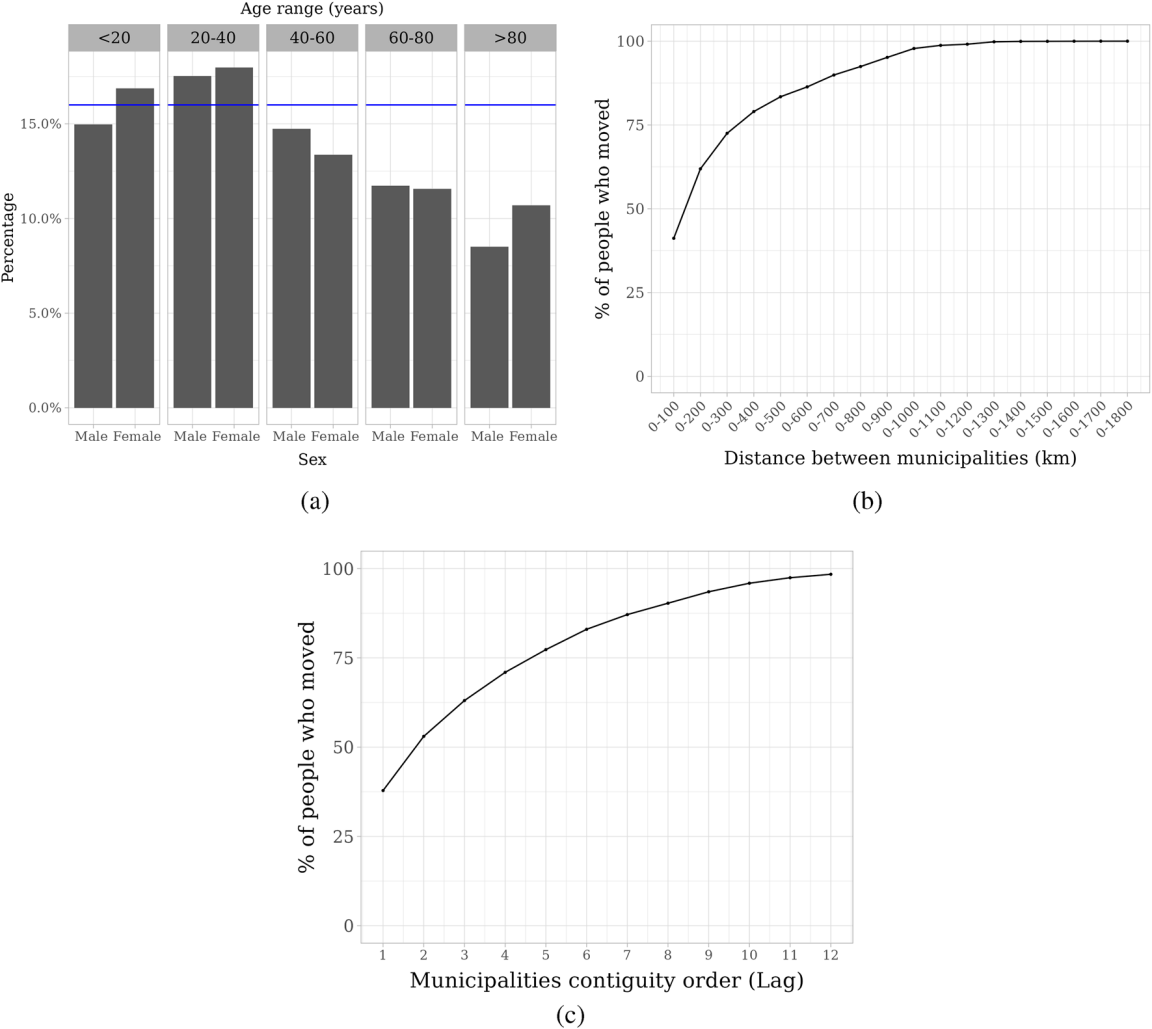
Cross-municipality migration rate stratified by demographics among people with laboratory-diagnosed tuberculosis (**a**) and stratified by distance (**b**) and contiguity between origin and destination municipalities (**c**) among people who moved. We calculated migration rate as the percentage of migrants among people with at least two clinic visits in each group. We defined migrants as people with laboratory-diagnosed tuberculosis who submitted tuberculosis test samples to clinics located in more than one municipality. We used laboratory tuberculosis test results, which were linked to identify individuals, from South Africa’s National Health Laboratory Service database to create a cohort (‘NHLS migration cohort’) to assess cross-municipality migration among people with laboratory-diagnosed tuberculosis. The blue line in (**a**) is migration rate in our migration cohort, distance in (**b**) was calculated as the geodesic distance between centroids of the origin and destination municipalities, and municipality contiguity order in (**c**) measures proximity between origin and destination municipalities based on kth order neighboring municipality.

The NHLS database included 921,888 people with laboratory-diagnosed TB with at least two clinic visits. Of these, 147,513 (16%) people submitted TB test samples to clinics located in more than one municipality, which, for this study, we assume indicates that they migrated between municipalities. Migration was slightly higher than average in females less than 20 years, and both males and females between 20–40 years respectively (*P*≤ 0.01 for all tests; Figure 1a). The median (IQR) distance between origin and destination municipalities was 304 (163 to 536) km. Of people who migrated between municipalities, 91,330 (62%) moved within 200 km (Figure 1b) and 55,836 (38%) moved between contiguous municipalities (Figure 1c). When stratified by TB type, similar results were observed among people with DS-TB and RR-TB, with higher migration rate among people with RR-TB than DS-TB (Table 1, and see Tables S2, S3 and Figures S2, S3, S4 in Appendix).

**Table 1:** Cross-municipality migration rate among people with laboratory-diagnosed TB stratified by TB type. We calculated migration rate as the percentage of migrants among people with at least two clinic visits in each group. We defined migrants as people with laboratory-diagnosed tuberculosis who submitted tuberculosis test samples to clinics located in more than one municipality. Data are laboratory tuberculosis test results, which were linked to identify individuals, from South Africa’s National Health Laboratory Service database. Patients test results were grouped into drug-susceptible and drug-resistant tuberculosis care episodes. People with no drug-resistant tuberculosis care episode were classified as drug-susceptible tuberculosis patients and people with at least one rifampicin-resistant tuberculosis care episode were classified as rifampicin-resistant tuberculosis patients.

TB type	Sample size	Number of migrants (%)
All TB	921,888	147,513 (16%)
Drug-susceptible TB	872,781	135,323 (15%)
Rifampicin-resistant TB	42,030	10,887 (26%)

**Figure 2:**
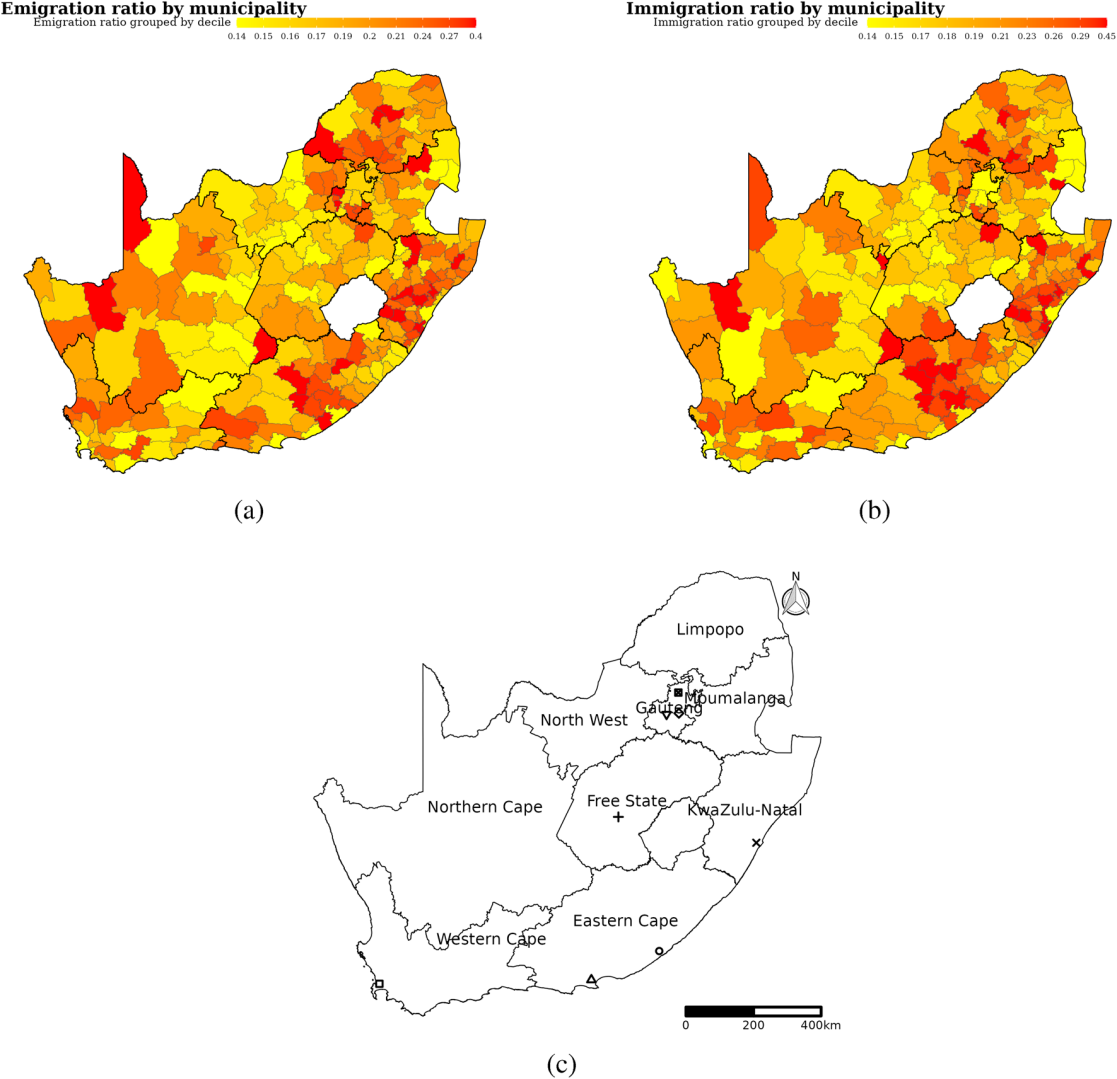
Emigration (**a**) and immigration ratios (**b**) by municipality among people with laboratory-diagnosed TB, and map of South Africa showing provincial boundaries and major cities (**c**). We calculated municipality-level emigration ratio as the share of outmigrants among people who initially visited a municipality, and immigration ratio as the share of in-migrants among all people who subsequently visited a municipality. We used laboratory tuberculosis test results, which were linked to identify individuals, from South Africa’s National Health Laboratory Service database to assess cross-municipality migration among people with laboratory-diagnosed tuberculosis. We defined migrants as people with laboratory-diagnosed tuberculosis who submitted tuberculosis test samples to clinics located in more than one municipality. The symbols in (**c**) are major cities in South Africa: Square, circle, triangle point up, plus, cross, diamond, triangle down, and square cross symbols are city of Cape town, Buffalo city, Nelson Mandela Bay, Mangaung, Ethekwini, Ekurhuleni, city of Johannesburg, and city of Tshwane respectively.

The immigration and emigration ratios by municipality among people with laboratory-diagnosed TB were 8 to 45% and 9 to 40% respectively. Higher immigration and emigration ratios were concentrated in eastern and northern provinces (KwaZulu-Natal, Gauteng, Limpopo, and Eastern Cape. see Fig. 2). Municipality-level and provincial-level immigration and emigration ratios stratified by DS-TB and RR-TB were estimated and presented in the Appendix (Table S4 and Figure S5).

### The role of migration in TB spread

**Figure 3:**
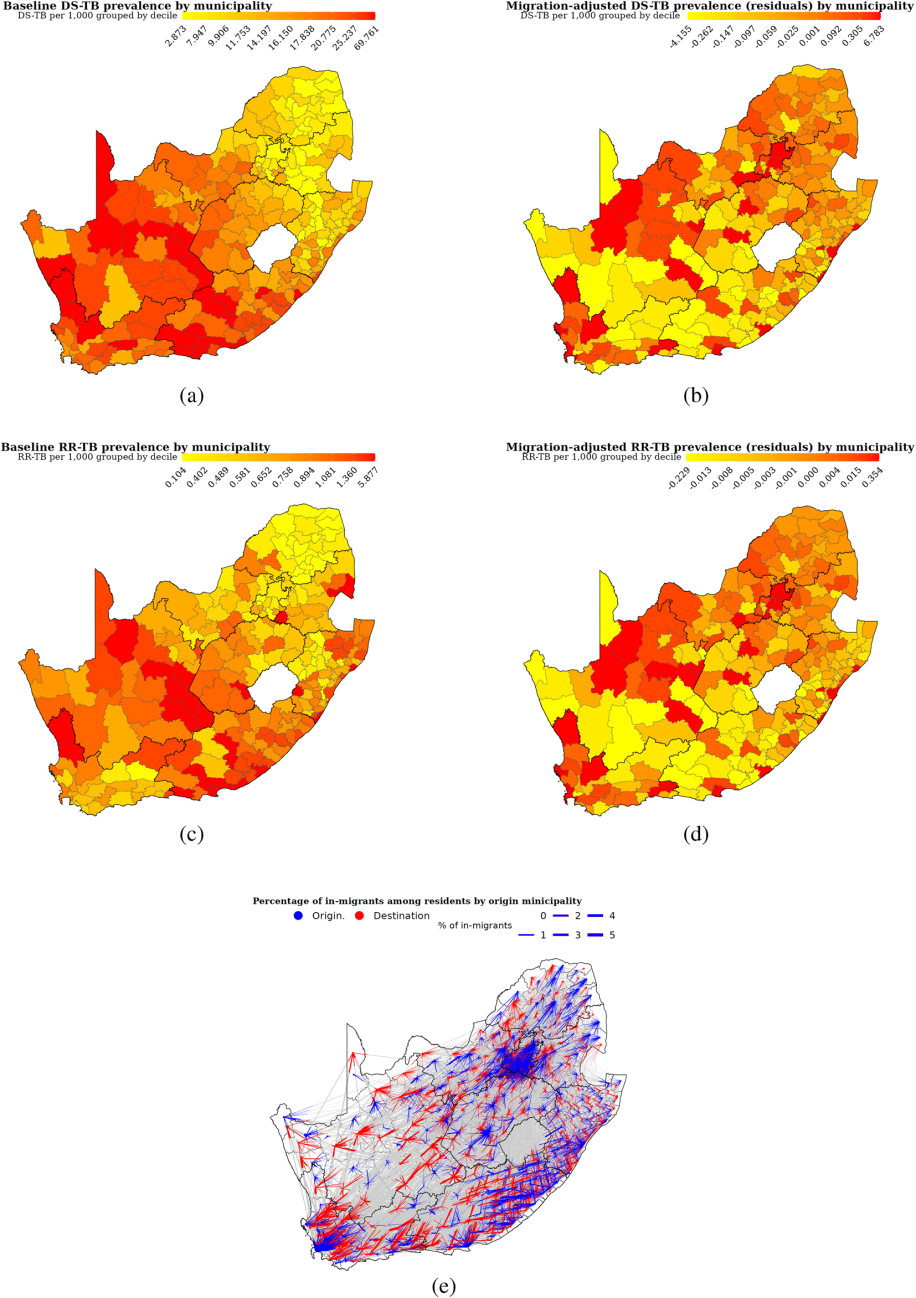
Baseline drug-susceptible tuberculosis prevalence (**a**), migration-adjusted drug-susceptible prevalence (**b**), baseline rifampicin-resistant tuberculosis prevalence (**c**), migration-adjusted rifampicin-resistant tuberculosis prevalence (**d**) by municipality, and cross-municipality migration rates (**e**). Migration-adjusted prevalence was presented as the residuals (difference between migration-adjusted and baseline prevalence) which we estimated using municipality-level tuberculosis prevalence from South Africa’s National Health Laboratory Service database and cross-municipality migration rates from the 2016 South African Community Survey.

We found that the median (IQR) baseline TB prevalence by municipality was 13.93 (9.00 to 18.83) per 1000 for DS-TB and 0.64 (0.44 to 0.97) per 1000 for RR-TB. The residual, i.e. the difference between migration-adjusted and baseline TB prevalence by municipality ranged from −4.15 to 6.78 per 1000 for DS-TB and −0.23 to 0.35 per 1,000 for RR-TB (Figure 3). The percentage change in baseline prevalence due to cross-municipality migration ranged from −13 to 44% (Figure S6 in Appendix).

We found that migration-adjusted DS-TB (RR-TB) prevalence was significantly associated with DS-TB (RR-TB) incidence two years later, even after controlling for unadjusted DS-TB (RR-TB) prevalence (Table 2). After controlling for baseline TB prevalence, each 1% increase in migration-adjusted DS-TB prevalence was associated with a 0.47% (95% CI: 0.03 to 0.90%) and 0.50% (95% CI: −0.27 to 1.27%) increase in annual DS-TB and RR-TB incidence in 2016 respectively. The predicted annual DS-TB (RR-TB) future incidence was 2.55% (2.78%) higher in the 90th than the 10th percentile of migration impact, which are places with higher and lower migration impact respectively (see section S3.1 in Appendix for details). However, the association between migration-adjusted prevalence and TB incidence one year later in 2015 was moderate, as each 1% increase in migration-adjusted TB prevalence was associated with a 0.26% (95% CI: −0.06 to 0.57%) increase in annual DS-TB after controlling for baseline prevalence. The association between migration-adjusted prevalence and RR-TB incidence one year later in 2015 was negative, as each 1% increase in migration-adjusted TB prevalence was associated with a −0.29% (95% CI: −0.54 to 0.31%) decrease in RR-TB incidence in 2015 (Table S6 in Appendix). Similar results were found when we formulated equations 1 and 2 as Poisson regression and Negative binomial models (see Tables S7 and S8 in Appendix). When cross-municipality migration rates were estimated from the NHLS data to calculate migration-adjusted prevalence, coefficients on migration-adjusted TB prevalence were attenuated towards zero and not statistically significant (see section S4 in Appendix for details).

**Table 2:** Association between future annual drug-susceptible tuberculosis (rifampicin-resistant tuberculosis) incidence and baseline drug-susceptible tuberculosis (rifampicin-resistant tuberculosis) prevalence and migration-adjusted drug-susceptible tuberculosis (rifampicin-resistant tuberculosis) prevalence. We estimated municipality-level (n = 234) migration-adjusted prevalence using tuberculosis burden from South Africa’s National Health Laboratory Service (NHLS) database and cross-municipality migration rates from the 2016 South African Community Survey. We have matched the survey data on the demographics among people with laboratory-diagnosed tuberculosis. We used prevalence:notification ratio from the 2018 South African national tuberculosis prevalence survey to convert laboratory-diagnosed tuberculosis cases by municipality into prevalence. Models 1 and 2 are regression models with baseline tuberculosis prevalence only and migration-adjusted prevalence only respectively, and model 3 has both predictors.

Outcome variable	DS-TB 2016					
	Model 1		Model 2		Model 3	
	Estimates	$95\% CI$	Estimates	$95\% CI$	Estimates	$95\% CI$
Unadjusted prevalence $\beta _1$	1.00	[0.91, 1.08]			0.53	[0.08, 0.99]
Adjusted prevalence $\beta _2$			1.00	[0.92, 1.08]	0.47	[0.03, 0.90]
Outcome variable	RR-TB 2016					
Unadjusted prevalence $\beta _1$	0.76	[0.63, 0.89]			0.25	[−0.53, 1.04]
Adjusted prevalence $\beta _2$			0.75	[0.63, 0.88]	0.50	[−0.27, 1.27]

## Discussion

We characterized and quantified the role of cross-municipality migration patterns in people with laboratory-diagnosed TB in TB spread. We found that 16% of people with at least two clinic visits submitted TB test samples in different municipalities (which was defined as migration), and most people migrated between nearby municipalities. Migration was higher in females less than 20 years and males and females between 20–40 years (Figure 1). In people with laboratory-diagnosed TB, migration was higher in eastern and northern provinces (Figure 2a,2b), and migration rate was higher among people with RR-TB than DS-TB (Table 1). Finally, we found that cross-municipality migration among people with laboratory-diagnosed TB were associated with future DS-TB and RR-TB incidence (Table 2).

High migration in people with TB could have important implications for care and management of TB. As more people with laboratory-diagnosed TB migrate between municipalities, it might be more difficult to ensure continuity of care, successful TB treatment, post-TB treatment and prevent TB recurrence. Naidoo and colleagues^22^ found that 29% of people who are diagnosed with TB do not receive or complete TB treatment in South Africa. Although this high loss in the TB care cascade in South Africa could be linked to failures in TB care, high cross-municipality migration could also play a role. TB treatment success rates in South Africa have been between 53–82% during the past 20 years, which is lower than the ≥90% target of the End TB strategy to be achieved by 2025^23^. Also, the TB recurrence rate was estimated to be 7.8% per year among gold miners and some urban communities in Cape Town^24,25^. Higher migration in people with TB can affect the effectiveness of TB prevention measures targeting people previously infected with TB. As such, access to TB data at the local level could facilitate longitudinal follow-up, help improve continuity of TB care and post-TB treatment to prevent recurrence.

Migration of people with TB could affect TB spread, and especially DR-TB spread as cross-municipality migration was higher among people with RR-TB than DS-TB. However, higher migration among people with RR-TB may reflect the DR-TB testing algorithm in different provinces, which often involves referral to different health facilities. Comparing across N=234 municipalities in South Africa, we found that migration-adjusted DS-TB (RR-TB) prevalence was associated with DS-TB (RR-TB) incidence two years later (Table 2). Municipalities that receive many migrants with TB had higher future incidence than municipalities that receive fewer migrants (average percent change was 2.5% and 2.8% per year for DS-TB and RR-TB respectively). Our results are consistent with a previous study, which showed that 84% of XDR TB transmission in KwaZulu-Natal province may be linked to cross-district migration^21,26^. To our knowledge, our work is the first study to report the association between cross-municipality migration patterns and municipality-level TB incidence using complete national data from South Africa’s public sector TB program.

Our study has limitations. First, we used cross-municipality migration rates from household survey data, which were matched to the NHLS TB cohort on age, sex, and origin municipality of TB patients, as a proxy for migration patterns among people with infectious TB. However, cross-municipality migration patterns in people who are infectious or vulnerable to TB infection would be more appropriate for investigating the impact of migration on TB spread. Unfortunately, migration data are lacking for these populations, and future studies could fill this gap. Second, we used national estimates of prevalence:notification ratios to convert diagnosed TB into prevalence by municipality. Prior studies suggest that case-detection rates may vary across health districts in South Africa, however, estimates of prevalence notification ratio by municipality are not available^27,28^. To address this concern, we conducted a sensitivity analysis and showed that our result may still hold if the municipality-level prevalence:notification ratios differ by reasonable order of magnitude (see section S5 in Appendix for details).

Although our study used long-term cross-municipality migration data to link TB spread and migration, seasonal migration during April and December holidays in South Africa could be important. We did not consider the impact of seasonal migration patterns in TB spread as estimates of seasonal migration rates are not available nationwide. Also, our data pertain to the time before the COVID-19 pandemic. Thus, our results might not be fully generalizable to pandemic-related lockdown periods or the post-pandemic era. However, migration patterns in the post-pandemic and pre-pandemic era are likely similar, therefore our conclusions may still hold going forward. Finally, our data are from before the introduction of GeneXpert Ultra. The inclusion of Ultra “trace” results could increase the number of people with diagnosed TB in our cohort. However, these additional cases are unlikely to change the migration patterns and the conclusions of our study.

Our work can help local and national tuberculosis programs and policy-makers predict future geographic patterns of TB disease. Spatial targeting of interventions using these types of data may be critical to improve the care of individual patients with tuberculosis as well as reduce population level transmission and prevalence.

## Methods

### Ethics statement

This research was approved by the Health Research Ethics Committee of the University of the Witwatersrand (no. M2111144) and Boston University Institutional Review Board (no. H-38441) for use of deidentified data with a waiver of consent. All methods were carried out in accordance with relevant guidelines and regulations.

### Study setting

South Africa is a high TB/HIV burden country, with an estimated TB incidence of 554/100,000 in 2020^29^. RR-TB was estimated to be 3.4% and 7.1% among new and previously treated TB cases respectively^23^. Until the August 2016 redistricting, South Africa was divided into nine provinces, 52 districts, and 234 metropolitan and local municipalities; we used these boundaries as most of our data were pre-August 2016.

### Data

### A. National health laboratory services (NHLS) TB cohort

We analyzed TB-related test records from the NHLS database between January 2011 and July 2017, including information on test dates, results, and the health facility where the sample was provided. Each record is a single laboratory TB test to confirm the presence of *Mycobacterium tuberculosis* (M.tb) or detect resistance to a TB drug (see section S1 in Appendix for details). Health facilities were geolocated to municipalities using their Geographic Information System coordinates, and facilities with missing coordinates were excluded. NHLS carries out 93% of all TB tests performed in South Africa^30^.

We developed and validated a record linkage algorithm to identify TB tests belonging to the same individual^31,32^. Our method replicates the approach previously used to construct a national HIV cohort from the NHLS database^33^. Using this validated unique identifier, we followed people with TB through care. During TB care, patients are ideally tested monthly and every three months for DS-TB and drug-resistant TB (DR-TB) respectively, but the treatment guideline varies by province^34^.

**Figure 4:**
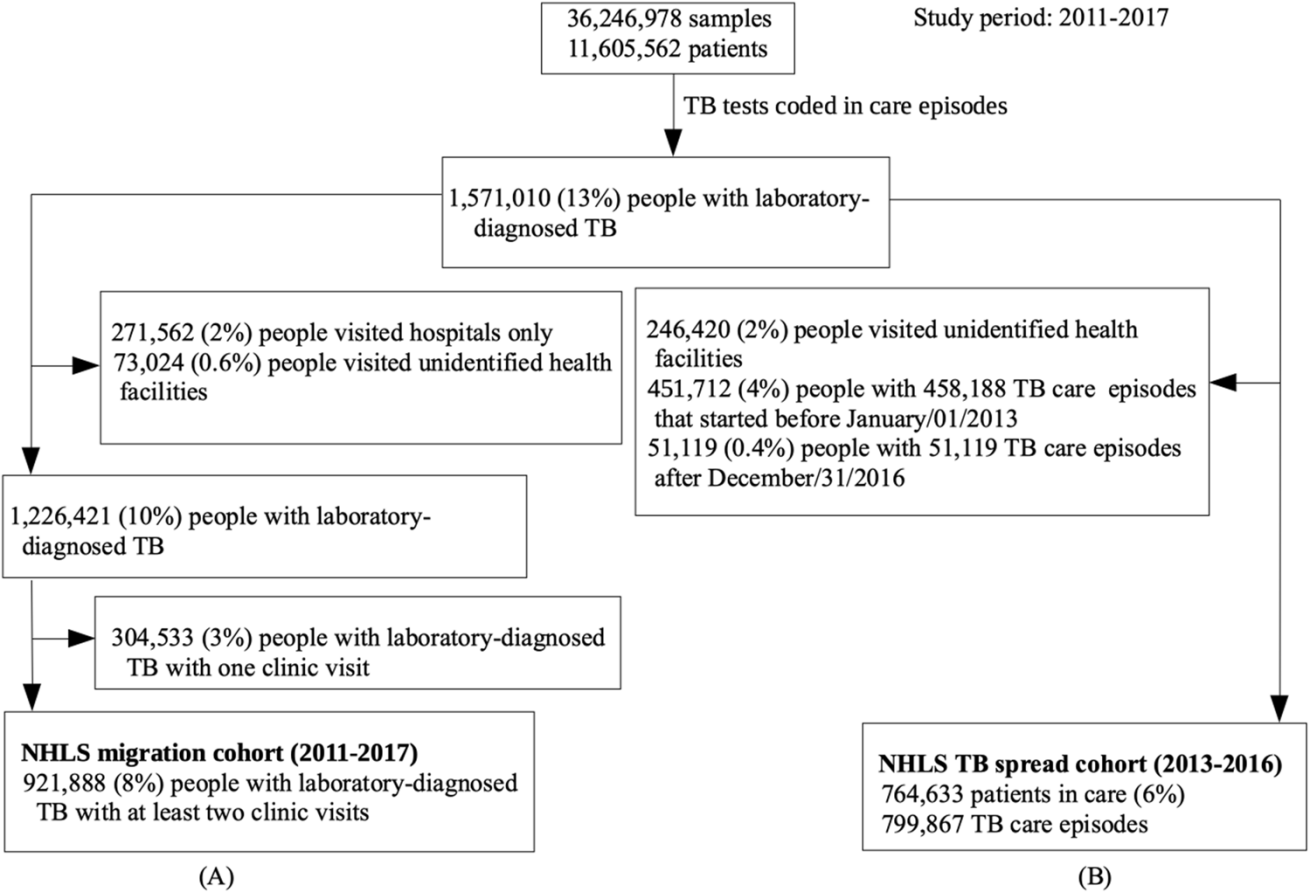
Flow diagram of our migration cohort (**A**) and tuberculosis spread cohort (**B**). The cohorts were created using tuberculosis laboratory results, which were linked to identify individuals, from South Africa’s National Health Laboratory Service database. We used the migration cohort to assess cross-municipality migration in people with laboratory-diagnosed tuberculosis and tuberculosis spread cohort to quantify the role of migration in tuberculosis spread.

We excluded TB test samples that were taken in hospital settings to separate mobility due to migration from mobility due to hospital referral because people may attend clinics near to their home but might travel longer distances for specialized in-hospital care. All the tests submitted within 30 days were considered as part of the same clinic visit, and we identified people with laboratory-diagnosed TB who had more than one clinic visit in the study period (“NHLS migration cohort” in Fig. 4A). For each individual, we defined age and sex in the NHLS migration cohort based on their first non-missing laboratory test, and all patients with at least one DR-TB care episode in the NHLS TB database were coded as DR-TB; otherwise they were coded as DS-TB.

### B. South African intercensal community survey data

The NHLS migration cohort enables us to describe migration patterns in people with laboratory-diagnosed TB. However, estimates of the cross-municipality migration rate using the NHLS migration cohort could be biased and less precise. First, some municipalities have small cohort size, and as such, estimates of migration rate for those municipalities are less precise (see section S6 in Appendix for details). Second, we have derived the municipality of residence of TB patients from the location of the facility where their laboratory tests were submitted, because the NHLS data do not report the municipality of residence of TB patients. As such, the municipality of origin and destination of TB patients could be misclassified and lead to biased and imprecise estimates of cross-municipality migration rates. We therefore estimated cross-municipality migration rates using a 5.8% public-use microdata sample of the 2016 South African Community Survey (“household survey data”), which is a national intercensal household survey conducted by the Government of South Africa (http://www.statssa.gov.za)^35^.

We extracted data on municipality of usual and prior residence for each survey participant, and we defined migration as an instance where a participant reported different municipalities of usual and previous residence. People with laboratory-diagnosed TB may have different migration patterns than the general population. For example, TB rates are higher among men and among working age people. To construct the cross-municipality migration rates, we adjusted the household survey data for age, sex, and municipality of origin, thus reweighting the household survey data so that the age and sex distribution matched that of people with TB in the NHLS migration cohort. However, factors other than sex and age like socio-economic status could bias our estimates of cross-municipality migration rates and this is a limitation of our study. We then used the matched household survey data to obtain estimates of cross-municipality migration rates, which we used to estimate migration-adjusted prevalence (see next section).

### Analysis

### A. Migration patterns in people with laboratory-diagnosed TB

We described migration patterns among people with laboratory-diagnosed TB with at least two clinic visits in the NHLS migration cohort. We defined migration as a person with laboratory-diagnosed TB who submitted samples in more than one municipality. To quantify migration rates by municipality, we identified the two most visited municipalities by each person with laboratory-diagnosed TB with at least two clinic visits in the NHLS dataset. We assumed that the municipality that was first visited among the two most visited municipalities is the person’s origin municipality, and the other municipality is the destination municipality. We calculated (1) the percentage of people with laboratory-diagnosed TB who moved, (2) the median distance travelled among people who moved (distance between centroids of the origin and destination municipalities), (3) emigration ratio (the share of outmigrants among people who initially visited a municipality), (4) immigration ratio (the share of in-migrants among all people who subsequently visited a municipality) by municipality.

### B. Analysis of the role of mobility in TB spread

To test our hypothesis that cross-municipality migration and TB spread are associated, we formulated a basic regression model, where future TB incidence depends on TB prevalence today. In the presence of migration, future incidence in a locality depends not just on local baseline TB prevalence but the number of people moving into that locality and TB prevalence in their locality of origin. We formalized this concept by constructing a measure of “migration-adjusted” TB prevalence and assessed whether migration is associated with future incidence after controlling for unadjusted baseline prevalence.

#### Municipality-level estimates of TB prevalence and incidence

We defined municipality i incidence in 2015 and 2016 as total number of people starting a TB care episode in that municipality in 2015 and 2016 in the NHLS database (Fig. 4B, “NHLS TB spread cohort”). We converted laboratory-diagnosed TB cases in 2013 and 2014 into prevalence using the prevalence:notification ratio by sex and age reported in the 2018 South African national TB prevalence survey (“prevalence survey data”) because many people with TB are undiagnosed or diagnosed clinically (see Table S5 in Appendix)^36^. We then estimated baseline TB prevalence by municipality as the average prevalence over 2013 and 2014. The prevalence:notification ratio may also vary by municipality because the different health districts in South Africa may not have the same health infrastructure for TB detection. As estimates of prevalence:notification ratios by municipality are not available, we conducted a sensitivity analysis to investigate the impact of differential TB detection at the municipality-level on our results.

#### Migration-adjusted TB prevalence

We defined “migration-adjusted” TB prevalence for municipality i as the weighted average of TB prevalence across all municipalities, with weights equal to the share of people with TB that originated in each municipality (see section S2 in Appendix for details). For municipalities that receive many migrants from high (low) prevalence areas, adjusted prevalence would be higher (lower) than unadjusted prevalence for that municipality. We used cross-municipality migration rates from the matched household survey data and municipality-level estimates of prevalence to estimates migration-adjusted prevalence in 2013 and 2014. We estimated migration-adjusted TB prevalence by municipality as the average migration-adjusted prevalence over 2013 and 2014. For completeness, we also estimated cross-municipality migration rates using the NHLS migration cohort. However, as noted above, the NHLS estimates are less precise, and as we are using cross-municipality migration rates as an explanatory variable, measurement error would bias the coefficient estimates towards zero^37^.

#### Regression models

We formulated the following linear regression models linking future incidence and current baseline and migration-adjusted prevalence,

1 $$\begin{aligned} log\bigg (\frac{I_{i, 2015}}{N_i}\bigg )= & {} \beta _{0} + \beta _{1} log\bigg (\frac{P_{i, 2013-2014}}{N_i}\bigg ) + \beta _{2} log\bigg (\frac{P'_{i, 2013-2014}}{N_i}\bigg ) + \varepsilon _i, \end{aligned}$$

2 $$\begin{aligned} log\bigg (\frac{I_{i, 2016}}{N_i}\bigg )= & {} \beta _{0} + \beta _{1} log\bigg (\frac{P_{i, 2013-2014}}{N_i}\bigg ) + \beta _{2} log\bigg (\frac{P'_{i, 2013-2014}}{N_i}\bigg ) + \varepsilon _i, \end{aligned}$$

where $N_i$, $I_{i, 2015}$, $I_{i, 2016}$, $P_{i,2013-2014}$, $P'_{i,2013-2014}$ and $\epsilon _{i}$ are population size, diagnosed TB incidence in 2015, diagnosed TB incidence in 2016, the baseline TB prevalence (unadjusted for migration) averaged over 2013 and 2014, migration-adjusted TB prevalence averaged over 2013 and 2014, and the error terms in municipality i respectively. The coefficients $\beta _0$, $\beta _1$ and $\beta _2$ are the intercept and the slopes associated with baseline TB prevalence ($P_{i,2013-2014}/N_i$) and migration-adjusted prevalence ($P'_{i,2013-2014}/N_i$) respectively. We presented linear regression models as this emerged from a simple epidemic model (Poisson and Negative binomial model formulations are presented in section 3 in Appendix).

Municipality population size ($N_i$) was retrieved from the 2011 South African national census data (http://www.statssa.gov.za). The coefficient of interest, $\beta _2$, is interpreted as the percent change in annual DS-TB and RR-TB by municipality for every 1% change in migration-adjusted DS-TB and RR-TB prevalence after controlling for baseline prevalence. We constructed 95% confidence intervals using heteroskedasticity-robust standard errors. We tested the null hypothesis that $\beta _{2} = 0$, which would imply that cross-municipality migration rates have no impact on DS-TB and RR-TB annual incidence after controlling for baseline prevalence. Finally, we stratified our analysis by urban and rural municipalities to investigate whether confounding variables like municipality population density and urban/rural status affect our results.

## Supplementary Information

Supplementary Information.

## Data Availability

Access to primary data is subject to restrictions owing to privacy and ethics policies set by the South African Government. Requests for access to the data can be made via the Office of Academic Affairs and Research at the National Health Laboratory Service through the AARMS research project application portal: https://aarms.nhls.ac.za/.
