# Supplementary material for: Cross-municipality migration and spread of tuberculosis in South Africa
Source: Sci Rep. 2023 Feb 15;13:2674. doi: 10.1038/s41598-023-29804-5 (PMC9930008; doi:10.1038/s41598-023-29804-5)
Supplement: Supplementary file 1 — Supplementary Information. [file 41598_2023_29804_MOESM1_ESM.pdf]

# Supplementary appendix: cross-municipality migration and spread of tuberculosis in South Africa

## Contents

**S1 Description of National Health Laboratory Services TB Cohort and definition of TB care episodes** **2**

**S2 Methods to investigate the role of migration in the spread of TB** **11**

S2.1 Estimation of baseline and migration-adjusted TB prevalence . . . . . . . . . . . . 11

S2.2 Note on the derivation of the regression model . . . . . . . . . . . . . . . . . . . . . 13

**S3 The role of cross-municipality migration in TB spread: cross-municipality migration rates were estimated using household survey data** **15**

S3.1 Impact of migration on future incidence . . . . . . . . . . . . . . . . . . . . . . 21

S3.2 The role of cross-municipality migration in TB spread stratified by urban and rural municipalities . . . . . . . . . . . . . . . . . . . . . . . . . . . . . . . . . . . 24

**S4 The role of cross-municipality migration in TB spread: cross-municipality migration rates were estimated using NHLS migration data** **26**

**S5 Sensitivity analysis of the effect of varying prevalence:notification ratio (P:N) by municipality** **31**

**S6 Correlation between migration rates from the HNLS and survey data** **36**

# S1. Description of National Health Laboratory Services TB Cohort and definition of TB care episodes

## National Health Laboratory Services (NHLS) TB data

We analyzed tuberculosis (TB) related test records from the National Health Laboratory Services (NHLS) database between January 2011 and July 2017, including information on test dates, results, and the health facility where the sample was provided. Health facilities were geolocated to municipalities using their Geographic Information System coordinates. NHLS carries out all TB tests in the public healthcare system, representing 93% of all TB tests performed in South Africa (1). Each NHLS database record is a single laboratory TB test to confirm the presence of *Mycobacterium tuberculosis* (M.tb) or detect resistance to a TB drug. Smear microscopy, culture with the mycobacterial growth indicator tube (Becton, Dickinson, and Company, https://www.bd.com) and Xpert MTB/RIF (Cepheid, https://www.cepheid.com) were used to detect the presence of M.tb. Drug susceptibility testing (DST), line probe assays (LPAs) with GenoType MTBDRplus (Hain Lifescience GmbH, https://www.hain-lifescience.de) and Xpert MTB/RIF were used to detect resistance to rifampicin (RR-TB) and other first-line drugs. We presented the percentage of people who were smear positive, culture positive and Xpert positive at initial diagnosis, which includes all TB test results that are within 14 days of the initial positive TB test result of a care episode (Table S1).

## Definition of care episodes and estimation of migration rate

We grouped all TB test results belonging to individuals into one or more TB care episodes and negative episodes. A TB care episode was defined as starting on the date of the initial positive TB test result in the NHLS dataset, which we assumed to be the diagnostic result for that care episode and included all subsequent tests (negative and positive) until the maximum likely

**Table S1:** Percentage of people who were smear positive, culture positive and Xpert positive at initial diagnosis by year in our cohorts (migration and TB spread cohorts from National Health Laboratory Services, NHLS, database). One person can have multiple care episodes or be positive on multiple tests at initial diagnosis.

| | | 2011 | 2012 | 2013 | 2014 | 2015 | 2016 | 2017 |
|---|---|---|---|---|---|---|---|---|
| NHLS migration cohort | Total care episodes | 177,722 | 160,434 | 163,589 | 164,569 | 161,914 | 145,341 | 31,072 |
| Total people: 921,888 | % smear positive | 76 | 66 | 48 | 44 | 43 | 45 | 47 |
| | % culture positive | 39 | 33 | 18 | 14 | 15 | 16 | 16 |
| | % Xpert positive | 7 | 28 | 69 | 85 | 86 | 86 | 84 |
| NHLS TB spread cohort | Total care episodes | | | 209,799 | 207,059 | 202,398 | 180,611 | |
| Total people: 764,633 | % smear positive | | | 46 | 41 | 40 | 42 | |
| | % culture positive | | | 18 | 14 | 14 | 16 | |
| | % Xpert positive | | | 67 | 83 | 85 | 85 | |

duration of a care episode, which we assumed to be 12 and 30 months for drug-susceptible TB (DS-TB) and drug-resistant TB (DR-TB, including rifampicin-resistant TB, isoniazid-resistant TB, and multi-drug-resistant TB) respectively. All TB care episodes with a positive RR-TB test result within 14 days of the initial positive TB test result were coded as RR-TB care episodes and DS-TB otherwise. Similar for isoniazid-resistant TB (INHR-TB, resistance to isoniazid), and multi-drug-resistant TB (MDR-TB, resistance to at least rifampicin and isoniazid). A new care episode was defined as a positive TB test result more than one year (two and half years for DR-TB) after the previous diagnostic positive test result. Negative episodes were those where the diagnostic test was TB-negative. We grouped TB test results into monthly clinic visits, where all the tests submitted within 30 days were part of the same clinic visit. Based on the above definitions, we identified people with laboratory-diagnosed TB who had more than one clinic visit in the study period “NHLS migration cohort" in Figure 4A in the main text) and their demographics are presented in Figure S1. All patients with no positive drug-resistant TB test result at initial diagnosis for all tests recorded in the NHLS TB database were coded as DS-TB; otherwise they were coded as RR-TB (people with at least one RR/MDR-TB care episode) or INHR-TB (all drug-resistant TB care episodes are INHR-TB).

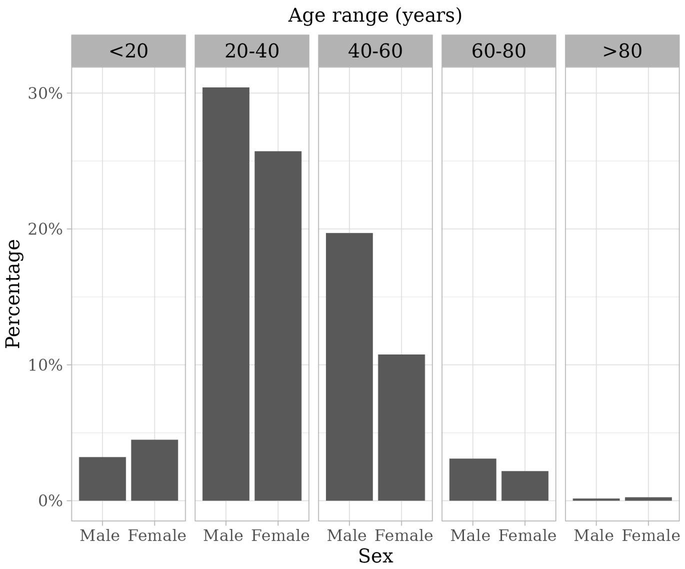


**Figure S1:** Sex and age distribution of people with laboratory-diagnosed TB who had at least 2 clinic visits in our migration cohort, created from National Health Laboratory Services (NHLS) TB database.

We assumed that people who submitted TB test samples to clinics located in more than one municipality were considered as people who migrated between municipalities. For each person who migrated, we identified the two most visited municipalities. We assumed that the municipality that was first visited among the two most visited municipalities is the person's origin municipality, and the other municipality is the destination municipality. We estimated migration rate among people with laboratory-diagnosed TB stratified by TB type (all TB, DS-TB, and DR-TB) and demographics (Table S2 and Figure S2). We found that cross-municipality migration was higher among people with RR-TB than DS-TB (26% vs 15%).

We investigated how far people with laboratory-diagnosed TB migrated by assessing the proximity by distance and contiguity between the origin and destination municipality for each person. Then, we calculated the minimum, the median (IQR), and the maximum distance between centroids of the origin and destination municipalities (Table S3). Finally, we calculated and graphed the percentage of people who migrated among people with all TB type, people with DS-TB, and people with DR-TB stratified by distance and contiguity between the origin and destination mu-

**Table S2:** Cross-municipality migration rate among people with laboratory-diagnosed TB (all TB), drug-susceptible TB (DS-TB), and rifampicin-resistant TB (RR-TB) stratified by demographics. Migration data are patient's clinic visits for TB testing from South Africa's National Health Laboratory Services TB database. Note: 10,443 (1%) people in our NHLS TB cohort have missing age or sex, and as such, the sum of people over the demographics is less than total people in our cohort.

| TB type and demographic characteristics | Sample size | Number of migrants (%) |
|---|---|---|
| All TB | 921,888 | 147,513 (16%) |
| Female < 20 | 40,918 | 6,901 (17%) |
| Male < 20 | 29,276 | 4,382 (15%) |
| Female 20 – 40 | 234,422 | 42,103 (18%) |
| Male 20 – 40 | 277,249 | 48,582 (17%) |
| Female 40 – 60 | 98,090 | 13,097 (13%) |
| Male 40 – 60 | 179,572 | 26,462 (15%) |
| Female 60 – 80 | 19,870 | 2,296 (12%) |
| Male 60 – 80 | 28,244 | 3,311 (12%) |
| Female > 80 | 2,321 | 248 (11%) |
| Male > 80 | 1,483 | 126 (8%) |
| Drug-susceptible TB | 872,781 | 135,323 (15%) |
| Female < 20 | 38,755 | 6,311 (16%) |
| Male < 20 | 27,896 | 4,064 (15%) |
| Female 20 – 40 | 220,056 | 38,242 (17%) |
| Male 20 – 40 | 262,842 | 44,782 (17%) |
| Female 40 – 60 | 92,842 | 12,010 (13%) |
| Male 40 – 60 | 170,356 | 24,252 (14%) |
| Female 60 – 80 | 19,185 | 2177 (11%) |
| Male 60 – 80 | 27,246 | 3,126 (11%) |
| Female > 80 | 2,255 | 233 (10%) |
| Male > 80 | 1,443 | 121 (8%) |
| Rifampicin-resistant TB | 42,030 | 10,887 (26%) |
| Female < 20 | 1,909 | 541 (28%) |
| Male < 20 | 1,380 | 296 (24%) |
| Female 20 – 40 | 12,351 | 3,465 (28%) |
| Male 20 – 40 | 12,304 | 3,366 (27%) |
| Female 40 – 60 | 4,479 | 983 (22%) |
| Male 40 – 60 | 7,818 | 1,949 (25%) |
| Female 60 – 80 | 587 | 109 (19%) |
| Male 60 – 80 | 838 | 161 (19%) |
| Female > 80 | 50 | 12 (24%) |
| Male > 80 | 35 | 5 (14%) |

nicipality (Figure S3).

People with RR-TB may have higher clinic visits than people with DS-TB, and as such, more likely to be observed in more than one municipality. We graphed the distribution of total number of clinic visits by patients, and we investigated migration rate among people with DS-TB and RR-TB

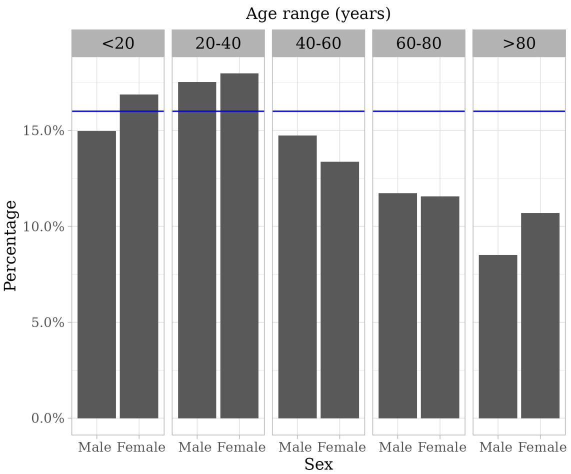


(a)

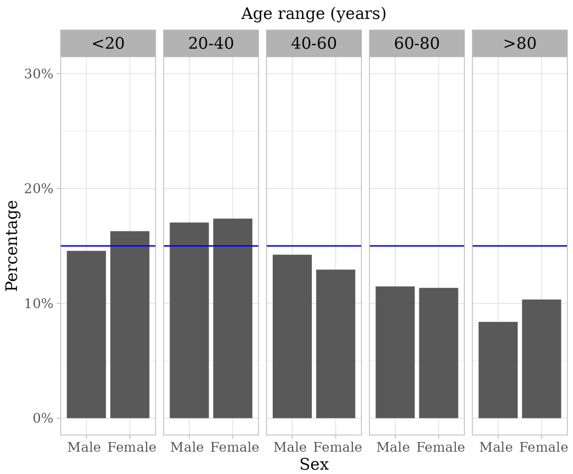


(b)

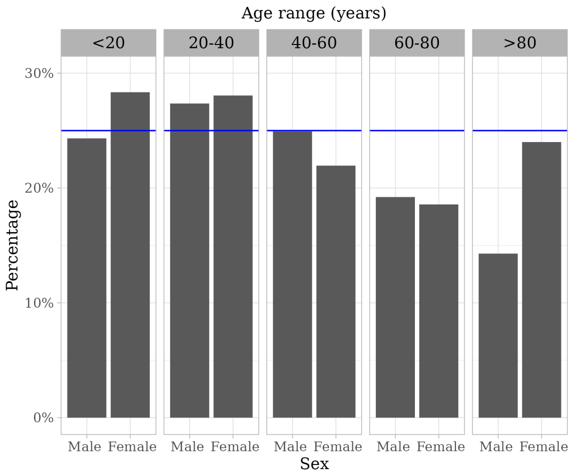


(c)

**Figure S2:** Cross-municipality migration rate among people with laboratory-diagnosed TB stratified by demographics for all TB (a), drug-susceptible TB (b), and rifampicin-resistant TB (c). Migration data are patient's clinic visits for TB testing from South Africa's National Health Laboratory Services TB database.

by range of clinic visits. We found that most of people had $<= 10$ clinic visits (Figure 4a), and migration rate was higher among people with RR-TB than DS-TB for clinic visits $<= 10$ (Figure 4b).

**Estimation of immigration and emigration ratios**

**Table S3:** Distance (in km) moved by people with laboratory-diagnosed TB stratified by TB type. Migration data are patient's clinic visits for TB testing from South Africa's National Health Laboratory Services TB database.

| TB type | Sample size | Minimum in km | Median (IQR) in km | Maximum in km |
|---|---|---|---|---|
| All TB types | 921,888 | 17 | 304 (163, 536) | 1735 |
| Drug-susceptible TB | 872,781 | 17 | 301 (161, 532) | 1735 |
| Rifampicin-resistant TB | 42,030 | 17 | 190 (98, 403) | 1735 |

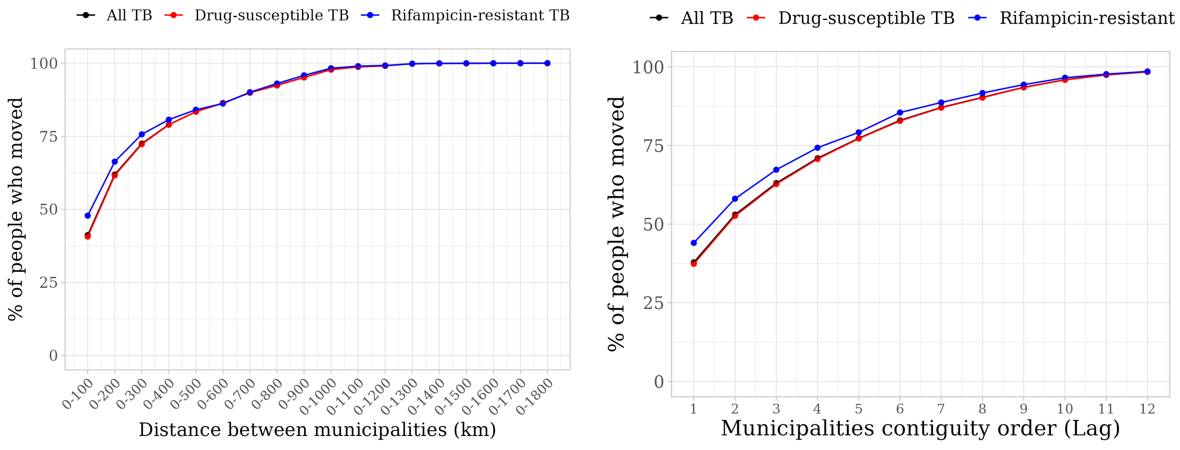


(a) (b)

**Figure S3:** Migration rate among people with laboratory-diagnosed TB (all TB, drug-susceptible TB, and drug-resistant TB) stratified by distance (a) and contiguity (b) between origin and destination municipalities. Migration data are patient's clinic visits for TB testing from South Africa's National Health Laboratory Services TB database.

We calculated municipality-level emigration ratio as the share of outmigrants among people who initially visited a municipality, and immigration ratio as the share of in-migrants among all people who subsequently visited a municipality. We averaged municipality-level emigration and immigration ratios to calculate provincial-level emigration and immigration ratio, stratified by TB type (Table S4 for provincial-level and Figure S5 for municipality-level migration ratios).

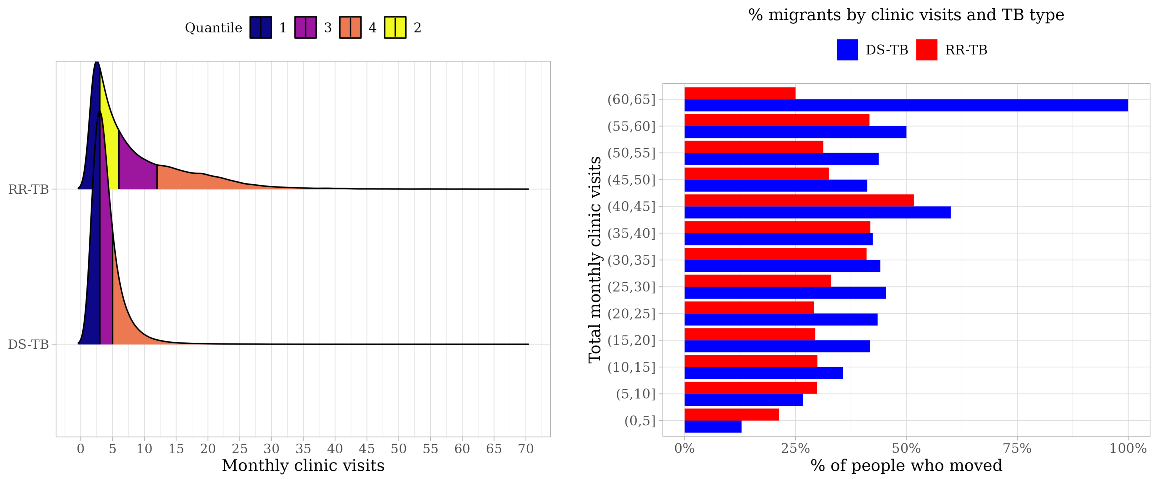


(a) (b)

**Figure S4:** Distribution of total clinic visits by TB type (a) and migration rate by TB type (b). Migration data are patient's clinic visits for TB testing from South Africa's National Health Laboratory Services TB database.

**Table S4:** Emigration and immigration ratio by province stratified by TB type. Emigration ratio is the share of outmigrants among people who were diagnosed in a municipality, and immigration ratio is the share of in-migrants among all people who subsequently visited a municipality. We averaged municipality-level emigration and immigration ratios to calculate provincial-level emigration and immigration ratio. Migration data are patient's clinic visits for TB testing from South Africa's National Health Laboratory Services TB database. Note: 10,443 (1%) people in our migration cohort have missing age or sex, and as such, the sum of people over the demographics is less than total people in our cohort.

| Provinces | TB type | Emigration ratio (%) | Immigration ratio (%) |
|---|---|---|---|
| Eastern Cape | all TB | 20.0 | 22.1 |
| | Drug- susceptible TB | 19.5 | 21.5 |
| | Rifampicin-resistant TB | 31.4 | 33.0 |
| Free State | all TB | 16.9 | 17.5 |
| | Drug- susceptible TB | 16.6 | 17.3 |
| | Rifampicin-resistant TB | 23.8 | 24.5 |
| Gauteng | all TB | 21.6 | 19.4 |
| | Drug- susceptible TB | 20.4 | 18.7 |
| | Rifampicin-resistant TB | 50.4 | 38.5 |
| KwaZulu-Natal | all TB | 22.3 | 23.4 |
| | Drug- susceptible TB | 21.4 | 22.7 |
| | Rifampicin-resistant TB | 41.4 | 38.7 |
| Limpopo | all TB | 21.2 | 22.3 |
| | Drug- susceptible TB | 20.5 | 21.5 |
| | Rifampicin-resistant TB | 40.7 | 35.2 |
| Mpumalanga | all TB | 17.5 | 18.4 |
| | Drug- susceptible TB | 16.9 | 17.8 |
| | Rifampicin-resistant TB | 28.1 | 28.2 |
| Northern Cape | all TB | 18.8 | 20.6 |
| | Drug- susceptible TB | 18.4 | 19.9 |
| | Rifampicin-resistant TB | 28.5 | 31.5 |
| North West | all TB | 16.3 | 17.1 |
| | Drug- susceptible TB | 16.0 | 16.9 |
| | Rifampicin-resistant TB | 24.0 | 25.3 |
| Western Cape | all TB | 17.8 | 18.9 |
| | Drug- susceptible TB | 17.5 | 18.5 |
| | Rifampicin-resistant TB | 26.1 | 26.9 |

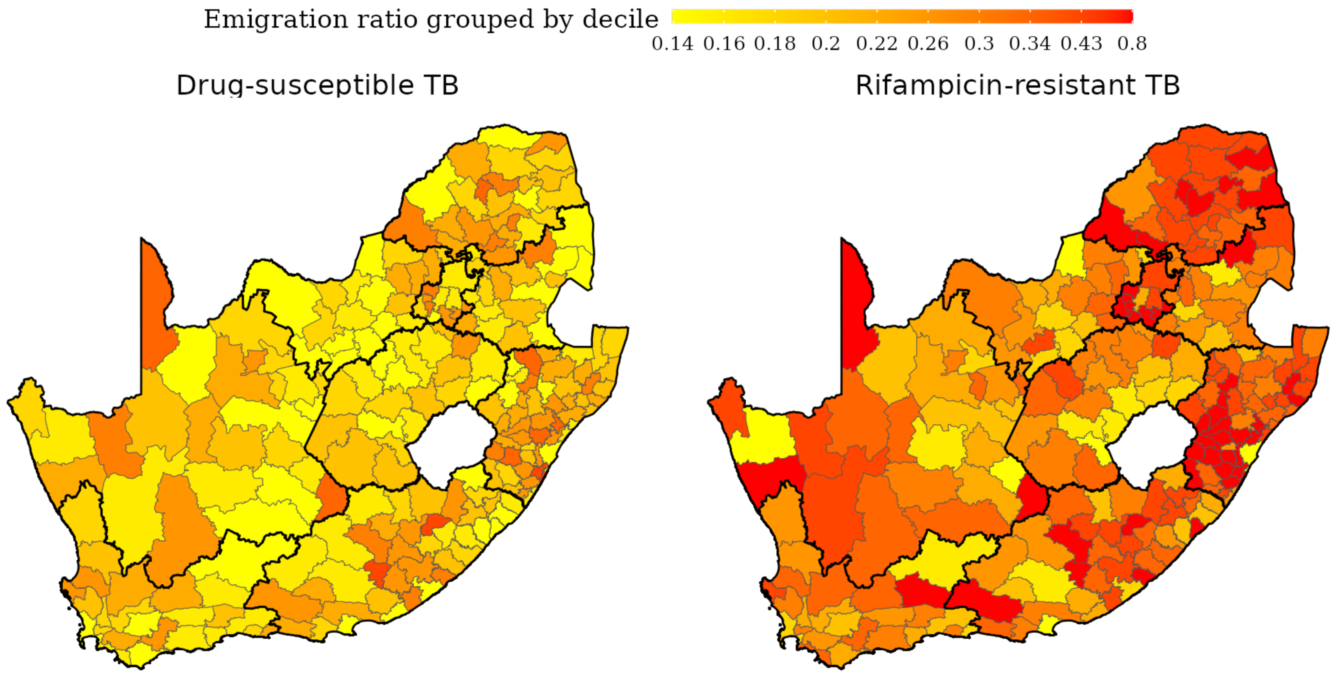


(a)

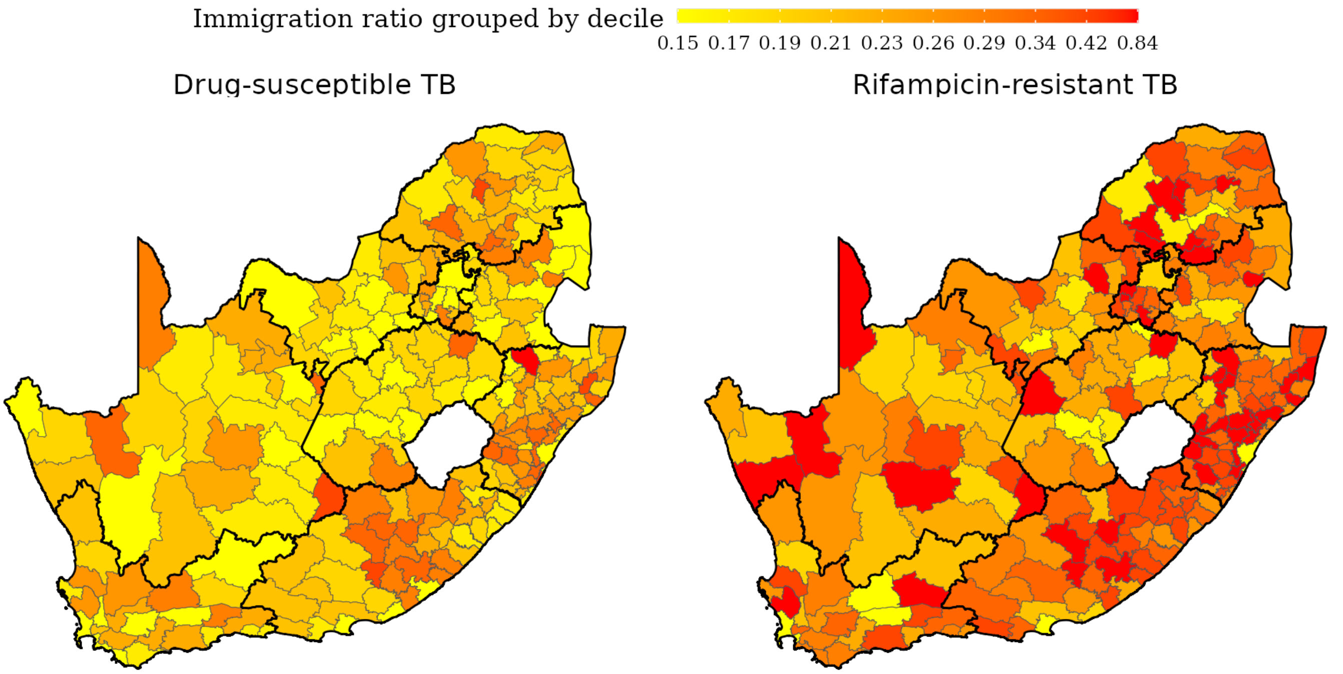


(b)

**Figure S5:** Municipality-level emigration (a) and immigration ratio (b) stratified by drug-susceptible and drug-resistant TB. Migration data are patient's clinic visits for TB testing from South Africa's National Health Laboratory Services TB database.

# S2. Methods to investigate the role of migration in the spread of TB

## S2.1. Estimation of baseline and migration-adjusted TB prevalence

TB prevalence in a locality during a time period is hard to estimate. People with laboratory-diagnosed TB in a locality at a given time are a fraction of all people who can transmit TB, as many people are undiagnosed or clinically diagnosed. We assumed that notified TB in a municipality $i$ at time $t$ is total number of people who started a TB care episode in that municipality at time $t$ (see “NHLS TB spread cohort", Figure 4B, in the main text). To estimate baseline prevalence, we converted total people with laboratory-diagnosed TB in each municipality in 2013 and 2014 into prevalence using the prevalence:notification ratio retrieved from the 2018 South African national TB prevalence survey (2). Then, we took the average over 2013 and 2014 to estimate baseline prevalence in each municipality. Our notification to prevalence conversion accounts for variation in the prevalence:notification ratio by sex and age because the age and sex structure, and as such, the proportion of people with TB who are undiagnosed may vary by municipality (Table S5).

**Table S5:** Prevalence:notification ratio by age and sex. Data were extracted from the 2018 South African TB prevalence survey in parenthesis by age and sex, and we calculated the ratio of prevalence:notification for a given sex and age group.

| Age group | 15-24 years (2.91) | 25-34 years (1.61) | 35-44 years (1.55) | 45-54 years (1.66) | 55-64 years (1.63) | $>$ 65 years (2.88) |
|---|---|---|---|---|---|---|
| Males (1.89) | 5.50 | 3.04 | 2.93 | 3.14 | 3.08 | 5.44 |
| Females (1.70) | 4.95 | 2.74 | 2.64 | 2.82 | 2.77 | 4.90 |

We defined prevalence in each municipality after migration as the sum of people with TB who did not migrate and people with TB who migrated from all the other municipalities. We defined

migration rate from municipality $j$ to $i$ as,

$$\frac{q_{ji}}{\sum_j q_{ji}}, \tag{S1}$$

where the numerator is the number of people who moved from municipality $j$ to $i$ and the denominator is total people in municipality $i$ after migration (this includes people who stayed in municipality $i$ and people who came from other municipalities). We estimated cross-municipality migration rates from our demographically-matched household survey data, where $q_{ji}$ is total people in municipality $i$ who responded that they lived in municipality $j$, and $\sum_j q_{ji}$ is total survey participants in municipality $i$. Let's define $P_{i,t}$ and $P'_{i,t}$ as baseline prevalence unadjusted for migration in municipality i at time t and migration-adjusted prevalence in municipality $i$ at time $t$ respectively. We estimated TB prevalence in municipality $i$ adjusted for cross-municipality migration in 2013 and 2014 as,

$$P'_{i,2013} = \frac{q_{ii}}{\sum_j q_{ji}} P_{i,2013} + \sum_{j \neq i} \left( \frac{q_{ji}}{\sum_j q_{ji}} P_{j,2013} \right), \tag{S2}$$

$$P'_{i,2014} = \frac{q_{ii}}{\sum_j q_{ji}} P_{i,2014} + \sum_{j \neq i} \left( \frac{q_{ji}}{\sum_j q_{ji}} P_{j,2014} \right). \tag{S3}$$

The first and the second terms in equations (S2 and S3) are people with TB who stayed in municipality $i$ and people with TB who migrated from other municipalities to municipality $i$ respectively. We estimated migration-adjusted prevalence by municipality as the average migration-adjusted prevalence over 2013 and 2014.

## S2.2. Note on the derivation of the regression model

We formulated the following equation to define the relation between TB incidence and prevalence in a municipality $i$,

$$\frac{dI_i}{dt} = \beta S_i \frac{P_i}{N_i}, \tag{S4}$$

where, $\beta$, $S_i$, $I_i$, and $N_i$ are TB transmission coefficient, the size of susceptible, new TB cases, prevalent TB cases, and population size in municipality $i$ respectively. Equation S4 means that future TB cases depends on prevalence today. Let's replace $\frac{dI_i}{dt}$, $S_i$, $P_i$, and $N_i$ with $I_{i,t+1}$, $S_{i,t}$, $P_{i,t}$, and $N_{i,t}$ respectively. We have,

$$I_{i,t+1} = \beta S_{i,t} \frac{P_{i,t}}{N_{i,t}}. \tag{S5}$$

Assuming that people with TB represent a small fraction of the entire population ($S_i \approx N_i$) and dividing both sides of equation S5 by $N$, we have,

$$\frac{I_{i,t+1}}{N_{i,t}} = \beta \frac{P_{i,t}}{N_{i,t}}. \tag{S6}$$

We log-transformed both sides of equation (S6) and we have,

$$log\left(\frac{I_{i,t+1}}{N_{i,t}}\right) = log(\beta) + log\left(\frac{P_{i,t}}{N_{i,t}}\right). \tag{S7}$$

We derived the following regression model from equation (S7),

$$log\left(\frac{I_{i,t+1}}{N_{i,t}}\right) = \beta_0 + \beta_1 log\left(\frac{P_{i,t}}{N_{i,t}}\right) + \varepsilon_i. \tag{S8}$$

We can write the same equation for migration-adjusted prevalence $\frac{P'_{i,t}}{N_{i,t}}$ as,

$$log\left(\frac{I_{i,t+1}}{N_{i,t}}\right) = \beta_0 + \beta_2 log\left(\frac{P'_{i,t}}{N_{i,t}}\right) + \varepsilon_i. \tag{S9}$$

where $\beta_0 = log(\beta)$, $\beta_1$, $\beta_2$, and $\varepsilon_i$ are the intercept, the slope associated with baseline prevalence, the slope associated with migration-adjusted prevalence, and the error term respectively.

Our goal is to investigate the association between future incidence and migration-adjusted prevalence after controlling for baseline prevalence. We know that variation in future incidence will partly be explained by variation in baseline prevalence and migration adjusted prevalence already include some aspects of baseline prevalence. In the absence of migration, baseline prevalence and migration-adjusted prevalence are the same. As such, we need to write our regression model to include both baseline prevalence and migration-adjusted prevalence to control for baseline prevalence.

$$log\left(\frac{I_{i,t+1}}{N_{i,t}}\right) = \beta_0 + \beta_1 log\left(\frac{P_{i,t}}{N_{i,t}}\right) + \beta_2 log\left(\frac{P'_{i,t}}{N_{i,t}}\right) + \varepsilon_i. \tag{S10}$$

Our coefficient of interest is $\beta_2$, which is interpreted as the percent change in future incidence by municipality for every 1% change in migration-adjusted prevalence after controlling for baseline prevalence. If cross-municipality migration and future incidence are associated then $\beta_2$ will be greater than zero after controlling for baseline prevalence.

For completeness, we also formalized equations 1 and 2 as Poisson and Negative binomial regression,

$$I_{i,2015} = log(N_i) + \beta_0 + \beta_1 log\left(\frac{P_{i,2013-2014}}{N_i}\right) + \beta_2 log\left(\frac{P'_{i,2013-2014}}{N_i}\right) + \varepsilon_i, \tag{S11}$$

$$I_{i,2016} = log(N_i) + \beta_0 + \beta_1 log\left(\frac{P_{i,2013-2014}}{N_i}\right) + \beta_2 log\left(\frac{P'_{i,2013-2014}}{N_i}\right) + \varepsilon_i, \tag{S12}$$

## S3. The role of cross-municipality migration in TB spread: cross-municipality migration rates were estimated using household survey data

We derived cross-municipality migration rates from demographically-matched household survey data to estimate migration-adjusted prevalence. To compare baseline prevalence (unadjusted for migration) and migration-adjusted prevalence, we calculated the percent change in baseline prevalence due to cross-municipality migration by municipality (see Figure S6). We investigated the association between migration-adjusted prevalence and future incidence using linear regression (Table S6), Poisson regression (Table S7), and Negative Binomial regression models (Table S8), and we stratified our analysis by DS-TB and RR-TB. Table S7 summarizes the results of Breusch-Pagan tests to investigate heteroskedasticity for our linear regression models, and Table S8 summarizes the results of equidispersion tests for our Poisson regression models. Equidispersion was tested using the function *dispersiontest* from AER package in R. $H_0$ assumes equal dispersion. For all tests, equal dispersion assumption was violated.

**Table S6:** Association between future annual DS-TB (RR-TB) incidence and baseline DS-TB (RR-TB) prevalence and migration-adjusted DS-TB (RR-TB) prevalence using linear regression formulation. Models 1 and 2 are equations (S11 and S12) with baseline TB prevalence only and migration-adjusted prevalence only respectively, and model 3 has both predictors. Estimated coefficients (95% CI) are percentage change in outcome variable for each 1% change in the explanatory variable.

| Outcome variable | DS-TB 2015 | | | | | |
|---|---|---|---|---|---|---|
| | Model 1 | | Model 2 | | Model 3 | |
| | Estimates | 95%*CI* | Estimates | 95%*CI* | Estimates | 95%*CI* |
| Unadjusted prevalence $\beta_1$ | 1.01 | [0.96,1.07] | | | 0.76 | [0.44,1.08] |
| Adjusted prevalence $\beta_2$ | | | 1.01 | [0.96,1.07] | 0.26 | [-0.06,0.57] |
| **Outcome variable** | **DS-TB 2016** | | | | | |
| Unadjusted prevalence $\beta_1$ | 1.00 | [0.91,1.08] | | | 0.53 | [0.08,0.99] |
| Adjusted prevalence $\beta_2$ | | | 1.00 | [0.92,1.08] | 0.47 | [0.03,0.90] |
| **Outcome variable** | **RR-TB 2015** | | | | | |
| Unadjusted prevalence $\beta_1$ | 0.77 | [0.69,0.86] | | | 1.07 | [0.17,1.97] |
| Adjusted prevalence $\beta_2$ | | | 0.77 | [0.68,0.86] | -0.29 | [-1.18,0.59] |
| **Outcome variable** | **RR-TB 2016** | | | | | |
| Unadjusted prevalence $\beta_1$ | 0.76 | [0.63,0.89] | | | 0.25 | [-0.53,1.04] |
| Adjusted prevalence $\beta_2$ | | | 0.75 | [0.63,0.88] | 0.50 | [-0.27,1.27] |

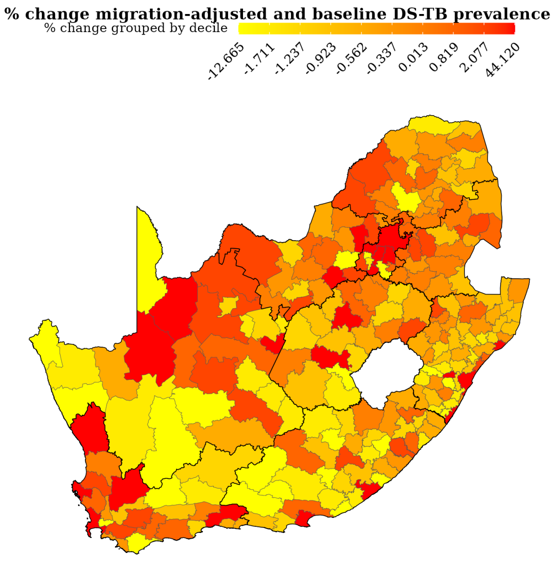


(a)

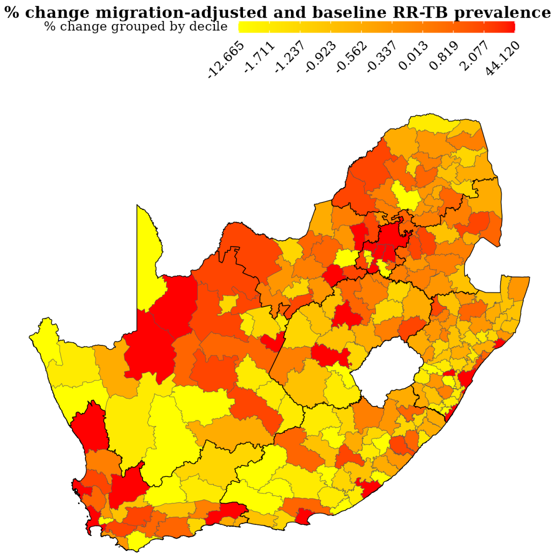


(b)

**Figure S6:** Percentage change in baseline prevalence due to cross-municipality migration for drug-susceptible tuberculosis (a) and rifampicin-resistant tuberculosis (b). Baseline TB prevalence is the average prevalence unadjusted for migration over 2013 and 2014, and migration-adjusted prevalence is the weighted average of TB prevalence across all municipalities, with weights equal to the share of people with TB that originated in each municipality. Laboratory-diagnosed TB cases by municipality from South Africa's National Health Laboratory Service (NHLS) TB database were converted into prevalence using prevalence:notification ratio from the 2018 South African national TB prevalence survey, and cross-municipality migration rates were derived from the 2016 South African household survey.

**Table S7:** Association between future annual DS-TB (RR-TB) incidence and baseline DS-TB (RR-TB) prevalence and migration-adjusted DS-TB (RR-TB) prevalence using Poisson regression model formulation. Models 1 and 2 are equations (S11 and S12) with baseline TB prevalence only and migration-adjusted prevalence only respectively, and model 3 has both predictors. Estimated (95%) coefficients were exponentiated. If $exp(\beta_2) > 1$ then $\beta_2 > 0$. $100 \times (exp(\beta_2) - 1)$ is the percentage change incidence for each 1 unit increase in $log(P^{'}_{2013-2014}/N)$.

| Outcome variable | DS-TB 2015 | | | | | |
|---|---|---|---|---|---|---|
| | Model 1 | | Model 2 | | Model 3 | |
| | Estimates | 95%*CI* | Estimates | 95%*CI* | Estimates | 95%*CI* |
| Unadjusted prevalence $\beta_1$ | 2.69 | [2.60,2.79] | | | 2.44 | [2.19,2.73] |
| Adjusted prevalence $\beta_2$ | | | 2.51 | [2.25,2.81] | 1.10 | [1.00,1.21] |
| **Outcome variable** | **DS-TB 2016** | | | | | |
| Unadjusted prevalence $\beta_1$ | 2.71 | [2.57,2.87] | | | 2.03 | [1.68,2.44] |
| Adjusted prevalence $\beta_2$ | | | 2.56 | [2.33,2.81] | 1.33 | [1·12,1.58] |
| **Outcome variable** | **RR-TB 2015** | | | | | |
| Unadjusted prevalence $\beta_1$ | 2.30 | [2.19,2.42] | | | 2.04 | [1.75,2.39] |
| Adjusted prevalence $\beta_2$ | | | 2.20 | [2.04,2.37] | 1.13 | [0.97,1.31] |
| **Outcome variable** | **RR-TB 2016** | | | | | |
| Unadjusted prevalence $\beta_1$ | 2.37 | [2.22,2.54] | | | 1.27 | [1.01,1.60] |
| Adjusted prevalence $\beta_2$ | | | 2.31 | [2.19,2.44] | 1.85 | [1.49,2.29] |

**Table S8:** Association between future annual DS-TB (RR-TB) incidence and baseline DS-TB (RR-TB) prevalence and migration-adjusted DS-TB (RR-TB) prevalence using Negative Binomial regression model. Models 1 and 2 are equations (S11 and S12) with baseline TB prevalence only and migration-adjusted prevalence only respectively, and model 3 has both predictors. Estimated (95%) coefficients were exponentiated. If $exp(\beta_2) > 1$ then $\beta_2 > 0$. $100 \times (exp(\beta_2) - 1)$ is the percentage change incidence for each 1 unit increase in $log(P'_{2013-2014}/N)$.

| Outcome variable | DS-TB 2015 | | | | | |
|---|---|---|---|---|---|---|
| | Model 1 | | Model 2 | | Model 3 | |
| | Estimates | 95%*CI* | Estimates | 95%*CI* | Estimates | 95%*CI* |
| Unadjusted prevalence $\beta_1$ | 2.75 | [2.63,2.88] | | | 2.26 | [1.71,2.98] |
| Adjusted prevalence $\beta_2$ | | | 2.75 | [2.63,2.87] | 1.22 | [0.93,1.61] |
| Outcome variable | DS-TB 2016 | | | | | |
| Unadjusted prevalence $\beta_1$ | 2.77 | [2.59,2.95] | | | 1.92 | [1.27,2.92] |
| Adjusted prevalence $\beta_2$ | | | 2.76 | [2.59,2.95] | 1.44 | [0.95,2.18] |
| Outcome variable | RR-TB 2015 | | | | | |
| Unadjusted prevalence $\beta_1$ | 2.24 | [2.10,2.40] | | | 2.51 | [1.64,3.86] |
| Adjusted prevalence $\beta_2$ | | | 2.23 | [2.08,2.38] | 0.89 | [0.58,1.37] |
| Outcome variable | RR-TB 2016 | | | | | |
| Unadjusted prevalence $\beta_1$ | 2.24 | [2.06,2.43] | | | 1.20 | [0.73,1.96] |
| Adjusted prevalence $\beta_2$ | | | 2.23 | [2.06,2.42] | 1.86 | [1.16,2.98] |

**Table S9:** Heteroskedasticity tests for models in Table S6 using Breusch-Pagan constant variance test. $H_0$: Heteroskedasticity is not present.

| Model (response) | BP-value | P-value | Decision |
|---|---|---|---|
| DS-TB 2015 | 3.63 | 0.16 | Heteroskedasticity is not present |
| DS-TB 2016 | 13.69 | 0.001 | Heteroskedasticity is present |
| RR-TB 2015 | 7.85 | 0.02 | Heteroskedasticity is present |
| RR-TB 2016 | 5.75 | 0.05 | Heteroskedasticity is not present |

**Table S10:** Equidispersion for each Poisson regression models in Table S7.

| Poisson model | Dispersion ($\phi$) | P-value | Decision |
|---|---|---|---|
| DS-TB 2015 | 8.84 | $1.5e-05$ | Equidispersion is not present |
| DS-TB 2016 | 16.28 | $1.1e-05$ | Equidispersion is not present |
| RR-TB 2015 | 1.47 | $8.6e-06$ | Equidispersion is not present |
| RR-TB 2016 | 1.65 | $4.5e-08$ | Equidispersion is not present |

## S3.1. Impact of migration on future incidence

We used our linear regression model parameter estimates to quantify the impact of cross-municipality migration on future incidence. First, we calculated the difference between migration-adjusted prevalence and baseline prevalence, the residuals, by municipality (Figure S7). Then, we found the 10th and the 90th percentiles of the residuals, which represent lower and higher impact of cross-municipality migration on TB prevalence respectively. We calculated the 10th (90th) percentile of migration-adjusted prevalence by municipality as the sum of municipality baseline prevalence and the 10th (90th) percentile of the residuals.

$$\frac{P'_{i,2013-2014,10th}}{N_i} = \frac{P_{i,2013-2014}}{N_i} + \text{10th percentile residuals}, \tag{S13}$$

and,

$$\frac{P'_{i,2013-2014,90th}}{N_i} = \frac{P_{i,2013-2014}}{N_i} + \text{90th percentile residuals}. \tag{S14}$$

Using our regression model (equation S12) and estimated coefficients ($\beta_0$, $\beta_1$, and $\beta_2$), we predicted future incidence by municipality in the 10th and 90th percentiles of migration impact. On linear scale, equation S12 is,

$$\frac{I_{i,2016,90th}}{N_i} = e^{(\beta_0)} \times \left(\frac{P_{i,2013-2014}}{N_i}\right)^{\beta_1} \times \left(\frac{P'_{i,2013-2014,90th}}{N_i}\right)^{\beta_2}. \tag{S15}$$

Finally, we calculated the percent change in future incidence due to migration in the 90th percentile ($\frac{I_{i,2016,90th}}{N_i}$) relative to the 10th percentile ($\frac{I_{i,2016,10th}}{N_i}$) of migration impact. We found that the percent change in future incidence in the 90th percentile compared to the 10th percentile of migration impact was on average 2.55% and 2.78% per year for drug-susceptible tuberculosis and drug-resistant tuberculosis respectively (Table S11).

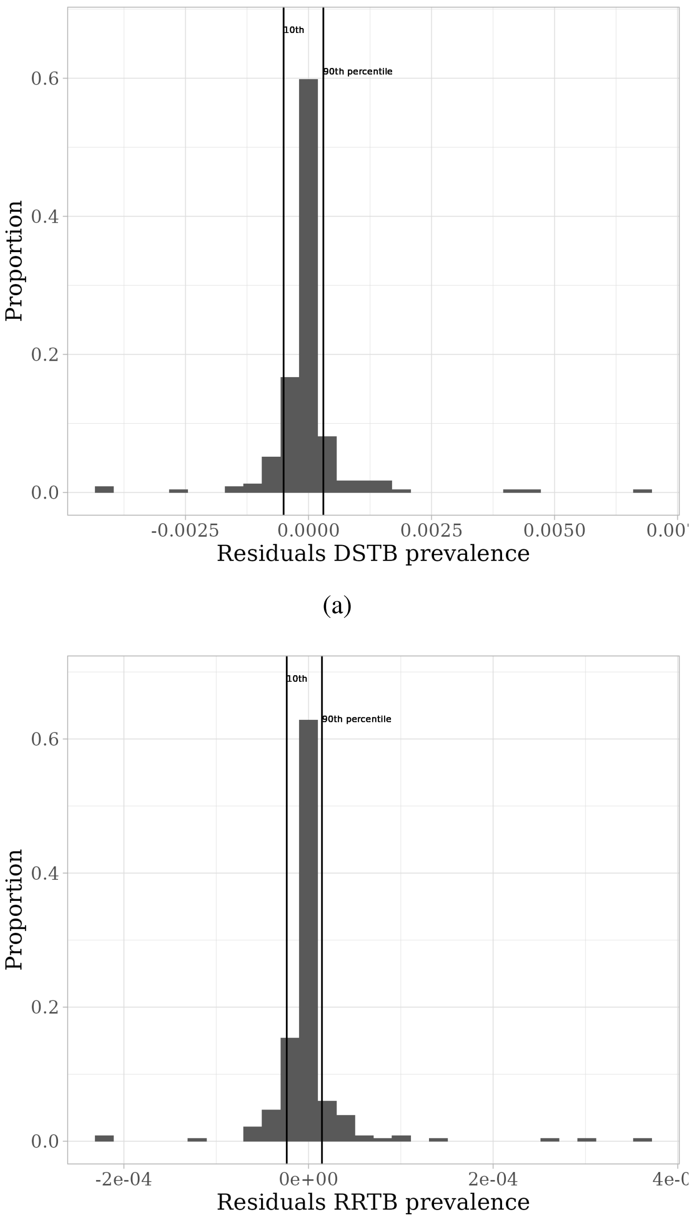


(a)

(b)

**Figure S7:** Distribution of residuals, i.e. the difference between migration-adjusted ($\frac{P_{i,2013-2014}}{N_i}$) and baseline TB prevalence ($\frac{P'_{i,2013-2014}}{N_i}$) by municipality for DSTB (a) and RRTB (b). The 10th and 90th percentiles are shown on both graphs.

**Table S11:** Predicted impact of cross-municipality migration on TB incidence.

| | 10th percentile incidence (per 100K) | 90th percentile incidence (per 100K) | Difference 90th and 10th percentile incidence (per 100K) | % change incidence 90th and 10th percentiles (per year) |
|---|---|---|---|---|
| Drug-susceptible TB 2016 | 389.36 | 399.27 | 9.92 | 2.55 |
| Rifampicin-resistant TB 2016 | 17.60 | 18.09 | 0.49 | 2.78 |

## S3.2. The role of cross-municipality migration in TB spread stratified by urban and rural municipalities

We used cross-municipality migration rates from demographically-matched household survey data to calculate migration-adjusted prevalence and investigate whether future incidence and migration-adjusted prevalence were associated for urban municipalities (Table S12) and rural municipalities (Table S13). Urban/rural classification was retrieved from the 2011 South African national census data (http://www.statssa.gov.za).

**Table S12:** Association between future annual DS-TB (RR-TB) incidence and baseline DS-TB (RR-TB) prevalence and migration-adjusted DS-TB (RR-TB) prevalence for urban municipalities. Models 1 and 2 are equations (1 and 2 in the main text) with baseline TB prevalence only and migration-adjusted prevalence only respectively, and model 3 has both predictors (Example equation 1: $log(I_{2015}/N_i) = \beta_0 + \beta_1 log(P_{2013-2014}/N) + \beta_2 log(P'_{2013-2014}/N)$). Estimated coefficients (95% CI) are percentage change in outcome variable for each 1% change in the explanatory variable.

| Outcome variable | DS-TB 2015 | | | | | |
|---|---|---|---|---|---|---|
| | Model 1 | | Model 2 | | Model 3 | |
| | Estimates | 95%*CI* | Estimates | 95%*CI* | Estimates | 95%*CI* |
| Unadjusted prevalence $\beta_1$ | 0.99 | [0.91,1.08] | | | 0.71 | [0.31,1.10] |
| Adjusted prevalence $\beta_2$ | | | 1.00 | [0.91,1.08] | 0.29 | [-0.12,0.70] |
| **Outcome variable** | **DS-TB 2016** | | | | | |
| Unadjusted prevalence $\beta_1$ | 0.96 | [0.82,1.10] | | | 0.59 | [0.06,1.12] |
| Adjusted prevalence $\beta_2$ | | | 0.96 | [0.83,1.10] | 0.38 | [−0.13,0.88] |
| **Outcome variable** | **RR-TB 2015** | | | | | |
| Unadjusted prevalence $\beta_1$ | 0.75 | [0.62,0.89] | | | 0.90 | [0.09,1.70] |
| Adjusted prevalence $\beta_2$ | | | 0.75 | [0.61,0.88] | -0.14 | [-0.93,0.64] |
| **Outcome variable** | **RR-TB 2016** | | | | | |
| Unadjusted prevalence $\beta_1$ | 0.65 | [0.44,0.85] | | | 0.42 | [-0.51,1.35] |
| Adjusted prevalence $\beta_2$ | | | 0.65 | [0.44,0.85] | 0.23 | [-0.68,1.13] |

**Table S13:** Association between future annual DS-TB (RR-TB) incidence and baseline DS-TB (RR-TB) prevalence and migration-adjusted DS-TB (RR-TB) prevalence for rural municipalities. Models 1 and 2 are equations (3 and 4) with baseline TB prevalence only and migration-adjusted prevalence only respectively, and model 3 has both predictors (equation 3: $log(I_{2015}/N_i) = \beta_0 + \beta_1 log(P_{2013-2014}/N) + \beta_2 log(P'_{2013-2014}/N)$). Both response and predictor variables were log-transformed. As such, the estimated coefficients (95% CI) are percentage change in outcome variable for each 1% change in the explanatory variable.

| Outcome variable | DS-TB 2015 | | | | | |
|---|---|---|---|---|---|---|
| | Model 1 | | Model 2 | | Model 3 | |
| | Estimates | 95%*CI* | Estimates | 95%*CI* | Estimates | 95%*CI* |
| Unadjusted prevalence $\beta_1$ | 0.80 | [0.66,0.94] | | | 1.37 | [-1.11,3.84] |
| Adjusted prevalence $\beta_2$ | | | 0.80 | [0.66,0.94] | -0.56 | [-3.07,1.94] |

| Outcome variable | DS-TB 2016 | | | | | |
|---|---|---|---|---|---|---|
| Unadjusted prevalence $\beta_1$ | 0.99 | [0.90,1.08] | | | 1.15 | [0.12,2.18] |
| Adjusted prevalence $\beta_2$ | | | 0.99 | [0.90,1.07] | -0.16 | [-1.19,0.86] |

| Outcome variable | RR-TB 2015 | | | | | |
|---|---|---|---|---|---|---|
| Unadjusted prevalence $\beta_1$ | 0.76 | [0.65,0.88] | | | 3.83 | [-0.45,8.10] |
| Adjusted prevalence $\beta_2$ | | | 0.76 | [0.65,0.88] | -3.07 | [-7.35,1.21] |

| Outcome variable | RR-TB 2016 | | | | | |
|---|---|---|---|---|---|---|
| Unadjusted prevalence $\beta_1$ | 0.80 | [0.66,0.94] | | | 1.37 | [-1.11,3.84] |
| Adjusted prevalence $\beta_2$ | | | 0.80 | [0.66,0.94] | -0.56 | [-3.07,1.94] |

## S4. The role of cross-municipality migration in TB spread: cross-municipality migration rates were estimated using NHLS migration data

We derived cross-municipality migration rates from NHLS migration cohort data to calculate migration-adjusted prevalence and investigate the association between future incidence and cross-municipality migration. We found that baseline TB prevalence ranged from 2.87 to 69.76 per 1,000 for DS-TB and 0.10 to 5.88 per 1,000 for RR-TB. The median (IQR) baseline TB prevalence by municipality was 13.93 (9.00 to 18.83) per 1,000 for DS-TB and 0.64 (0.44 to 0.97) per 1,000 for RR-TB. The residual, i.e. the difference between migration-adjusted and baseline TB prevalence by municipality ranged from -20.79 to 47.73 per 1,000 and the median (IQR) was -0.76 (-1.69 to 0.28) per 1,000 for DS-TB. The residual by municipality ranged from -1.75 to 3.06 per 1,000 and the median (IQR) was -0.03 (-1.09 to 0.01) per 1,000 for RR-TB (Fig S8). We investigated the association between migration-adjusted prevalence and future incidence using linear regression (Table S14), Poisson regression (Table S15), and Negative Binomial regression models (Table S16). Table S17 summarizes the results of equidispersion tests for each Poisson regression model. Equidispersion was tested using the function *dispersiontest* from AER package in R. $H_0$ assumes equal dispersion. For all equations, equal dispersion assumption was violated.

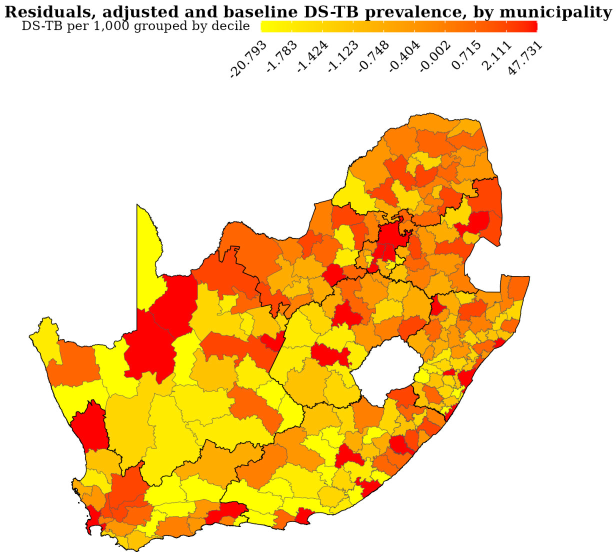


(a)

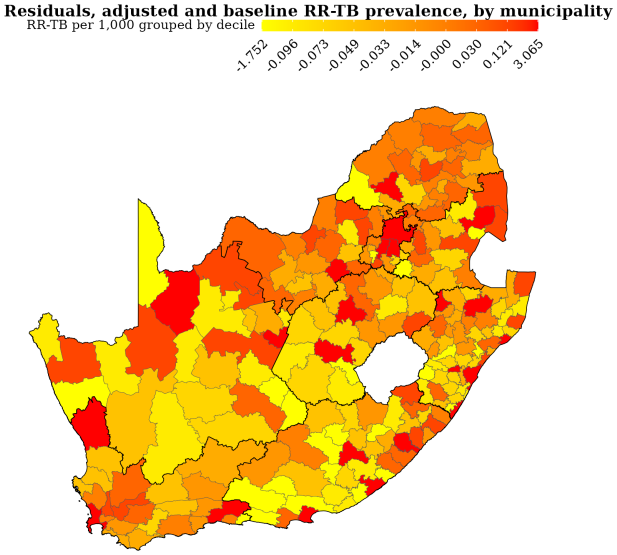


(b)

**Figure S8:** Residual, i.e. the difference between migration-adjusted and baseline TB prevalence by municipality for DS-TB (a) and RR-TB (b). Cross-municipality migration rate were derived from NHLS migration cohort.

**Table S14:** Association between future annual DS-TB (RR-TB) incidence and baseline DS-TB (RR-TB) prevalence and migration-adjusted DS-TB (RR-TB) prevalence using linear regression formulation. Models 1 and 2 are equations (S11 and S12) with baseline TB prevalence only and migration-adjusted prevalence only respectively, and model 3 has both predictors. Estimated coefficients (95% CI) are percentage change in outcome variable for each 1% change in the explanatory variable. Cross-municipality migration rate were derived from NHLS migration cohort.

| Outcome variable | DS-TB 2015 | | | | | |
|---|---|---|---|---|---|---|
| | Model 1 | | Model 2 | | Model 3 | |
| | Estimates | 95%*CI* | Estimates | 95%*CI* | Estimates | 95%*CI* |
| Unadjusted prevalence $\beta_1$ | 1.01 | [0.96,1.07] | | | 0.90 | [0.79,1.02] |
| Adjusted prevalence $\beta_2$ | | | 0.89 | [0.80,0.97] | 0.11 | [0.02,0.21] |
| **Outcome variable** | **DS-TB 2016** | | | | | |
| Unadjusted prevalence $\beta_1$ | 1.00 | [0.91,1.08] | | | 0.83 | [0.64,1.02] |
| Adjusted prevalence $\beta_2$ | | | 0.88 | [0.79,0.96] | 0.17 | [0.03,0.30] |
| **Outcome variable** | **RR-TB 2015** | | | | | |
| Unadjusted prevalence $\beta_1$ | 0.77 | [0.69,0.86] | | | 0.75 | [0.57,0.93] |
| Adjusted prevalence $\beta_2$ | | | 0.69 | [0.60,0.78] | 0.02 | [-0.13,0.18] |
| **Outcome variable** | **RR-TB 2016** | | | | | |
| Unadjusted prevalence $\beta_1$ | 0.76 | [0.63,0.89] | | | 0.57 | [0.33,0.81] |
| Adjusted prevalence $\beta_2$ | | | 0.69 | [0.57,0.80] | 0.18 | [-0.01,0.38] |

**Table S15:** Association between future annual DS-TB (RR-TB) incidence and baseline DS-TB (RR-TB) prevalence and migration-adjusted DS-TB (RR-TB) prevalence using Poisson regression model formulation. Models 1 and 2 are equations (S11 and S12) with baseline TB prevalence only and migration-adjusted prevalence only respectively, and model 3 has both predictors. Estimated (95%) coefficients were exponentiated. If $exp(\beta_2) > 1$ then $\beta_2 > 0$. $100 \times (exp(\beta_2) - 1)$ is the percentage change incidence for each 1 unit increase in $log(P'_{2013-2014}/N)$. Cross-municipality migration rate were derived from NHLS migration cohort.

| Outcome variable | DS-TB 2015 | | | | | |
|---|---|---|---|---|---|---|
| | Model 1 | | Model 2 | | Model 3 | |
| | Estimates | 95%*CI* | Estimates | 95%*CI* | Estimates | 95%*CI* |
| Unadjusted prevalence $\beta_1$ | 2.69 | [2.60,2.79] | | | 2.63 | [2.49,2.77] |
| Adjusted prevalence $\beta_2$ | | | 1.58 | [1.25,1.99] | 1.02 | [0.98,1.07] |
| **Outcome variable** | **DS-TB 2016** | | | | | |
| Unadjusted prevalence $\beta_1$ | 2.71 | [2.57,2.87] | | | 2.54 | [2.32,2.79] |
| Adjusted prevalence $\beta_2$ | | | 1.61 | [1.27,2.04] | 1.07 | [0·98,1.16] |
| **Outcome variable** | **RR-TB 2015** | | | | | |
| Unadjusted prevalence $\beta_1$ | 2.30 | [2.19,2.42] | | | 2.24 | [2.10,2.39] |
| Adjusted prevalence $\beta_2$ | | | 1.57 | [1.30,1.89] | 1.01 | [0.98,1.07] |
| **Outcome variable** | **RR-TB 2016** | | | | | |
| Unadjusted prevalence $\beta_1$ | 2.37 | [2.22,2.54] | | | 2.03 | [1.84,2.24] |
| Adjusted prevalence $\beta_2$ | | | 2.66 | [1.42,1.95] | 1.15 | [1.08,1.22] |

**Table S16:** Association between future annual DS-TB (RR-TB) incidence and baseline DS-TB (RR-TB) prevalence and migration-adjusted DS-TB (RR-TB) prevalence using Negative Binomial regression model. Models 1 and 2 are equations (S11 and S12) with baseline TB prevalence only and migration-adjusted prevalence only respectively, and model 3 has both predictors. Estimated (95%) coefficients were exponentiated. If $exp(\beta_2) > 1$ then $\beta_2 > 0$. $100 \times (exp(\beta_2) - 1)$ is the percentage change incidence for each 1 unit increase in $log(P^{'}_{2013-2014}/N)$. Cross-municipality migration rate were derived from NHLS migration cohort.

| Outcome variable | DS-TB 2015 | | | | | |
|---|---|---|---|---|---|---|
| | Model 1 | | Model 2 | | Model 3 | |
| | Estimates | 95%*CI* | Estimates | 95%*CI* | Estimates | 95%*CI* |
| Unadjusted prevalence $\beta_1$ | 2.75 | [2.63,2.88] | | | 2.51 | [2.28,2.77] |
| Adjusted prevalence $\beta_2$ | | | 2.47 | [2.31,2.64] | 1.10 | [1.01,1.19] |
| Outcome variable | DS-TB 2016 | | | | | |
| Unadjusted prevalence $\beta_1$ | 2.77 | [2.59,2.95] | | | 2.42 | [2.09,2.79] |
| Adjusted prevalence $\beta_2$ | | | 2.47 | [2.30,2.66] | 1.14 | [1.02,1.28] |
| Outcome variable | RR-TB 2015 | | | | | |
| Unadjusted prevalence $\beta_1$ | 2.24 | [2.10,2.40] | | | 2.27 | [2.03,2.54] |
| Adjusted prevalence $\beta_2$ | | | 2.02 | [1.85,2.20] | 0.99 | [0.90,1.08] |
| Outcome variable | RR-TB 2016 | | | | | |
| Unadjusted prevalence $\beta_1$ | 2.24 | [2.06,2.43] | | | 1.95 | [1.66,2.29] |
| Adjusted prevalence $\beta_2$ | | | 2.03 | [1.87,2.20] | 1.14 | [1.01,1.29] |

**Table S17:** Equidispersion for each Poisson regression model in Table S15.

| Poisson model | Dispersion ($\phi$) | P-value | Decision |
|---|---|---|---|
| $log(DS-TB2015/N_i) \sim log(P_{i,2013-2014}/N_i) + log(P'_{i,2013-2014}/N_i) + \varepsilon_i$ | 8.84 | $1.2e-05$ | Equidispersion is not present |
| $log(DS-TB2016/N_i) \sim log(P_{i,2013-2014}/N_i) + log(P'_{i,2013-2014}/N_i) + \varepsilon_i$ | 16.40 | 2.23 $1.2e-05$ | Equidispersion is not present |
| $log(RR-TB2015/N_i) \sim log(P_{i,2013-2014}/N_i) + log(P'_{i,2013-2014}/N_i) + \varepsilon_i$ | 1.47 | $8.6e-06$ | Equidispersion is not present |
| $log(RR-TB2016/N_i) \sim log(P_{i,2013-2014}/N_i) + log(P'_{i,2013-2014}/N_i) + \varepsilon_i$ | 1.68 | $3.3e-08$ | Equidispersion is not present |

# S5. Sensitivity analysis of the effect of varying prevalence:notification ratio (P:N) by municipality

In investigating the effect of cross-municipality migration in TB spread, we assumed that the prevalence:notification ratio by sex and age is the same for all municipalities (uniform ratio). However, the prevalence:notification ratio may vary by municipality (non-uniform ratio) because the different health districts in South Africa do not have the same health infrastructure for TB detection (3; 4). Using national estimates of prevalence:notification ratio by age and sex to convert diagnosed TB into prevalence for all municipalities may underestimate or overestimate prevalence in some municipalities and affect our results. We conducted a sensitivity analysis to investigate whether our results still hold with the assumption that prevalence:notification ratio by age and sex differs among municipalities. We constructed three range of values of prevalence:notification ratio by age and sex, $[0.75 \times P:N, 1.25 \times P:N]$, $[0.5 \times P:N, 1.5 \times P:N]$ and $[1, 2 \times P:N]$, where the first and second values are the lower and upper bounds of the range, and $P:N$ is national estimate of prevalence:notification ratio by age and sex which was retrieved from the 2018 South African national TB prevalence survey (2). The three range of values represent small medium and large difference in municipality-level estimates compared to national-level estimates of prevalence:notification ratio. For each range we run 30 iterations of our linear regression model (equations S11 and S12), where municipality-level prevalence:notification ratio by age and sex is randomly sampled from the range (Figures S9-S12).

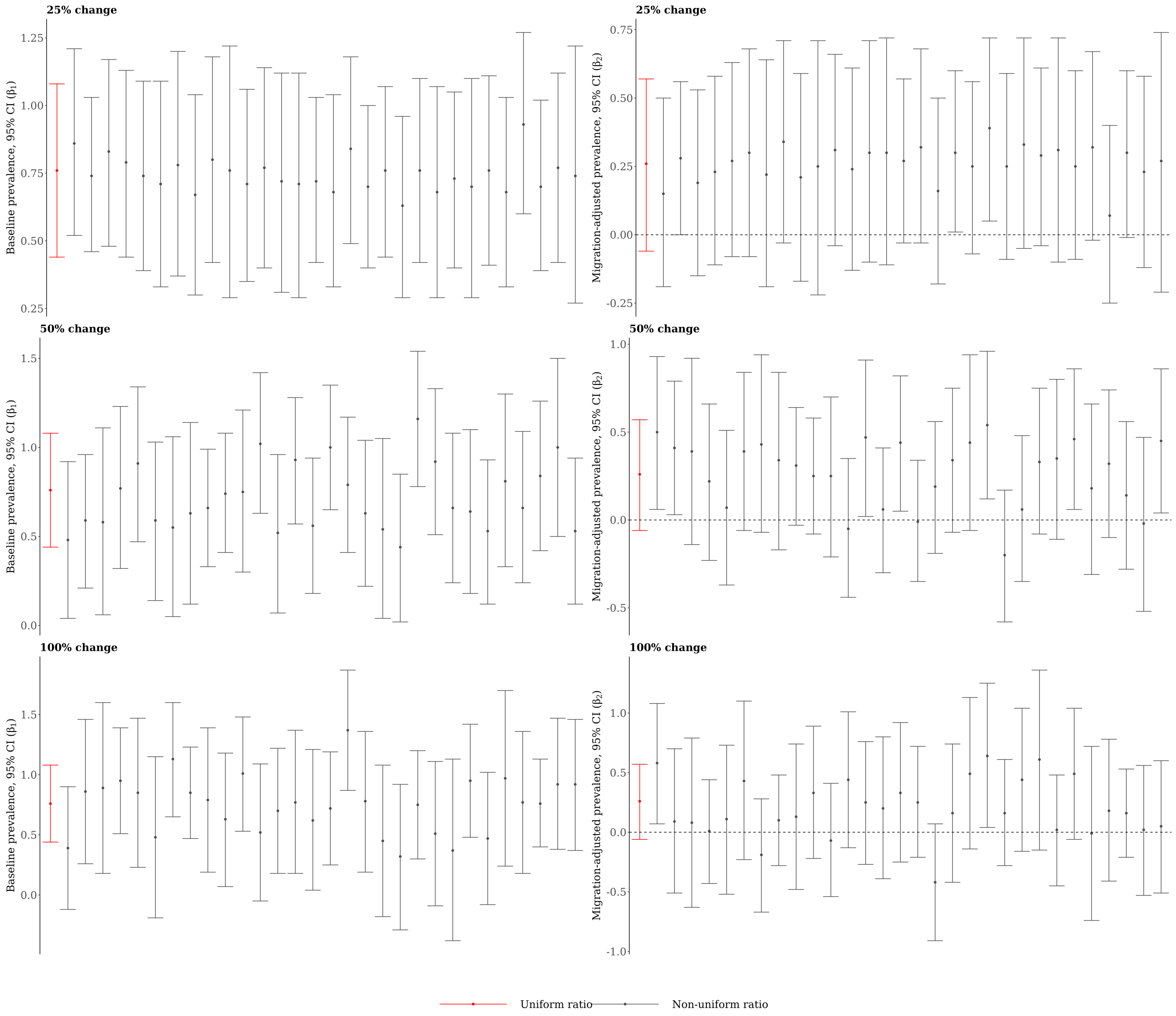


**Figure S9:** Estimated regression parameters and 95% CI for our linear regression model (equation S11, DS-TB in 2015) with White-Huber robust standard errors (heteroskedasticity-consistent standard errors) where prevalence:notification ratio differs by age, sex, and municipality. Prevalence notification ratios (30 iterations) were randomly sampled from for the ranges $[0.75 \times P : N, 1.25 \times P : N]$, $[0.5 \times P : N, 1.5 \times P : N]$ and $[1, 2 \times P : N]$, where $P : N$ is national estimate of prevalence:notification ratio, for the first, second, and third row respectively. Estimated regression parameters with national estimates of prevalence:notification ratio (uniform ratio) are in red and municipality-level prevalence:notification ratio (non-uniform ratio) are in grey.

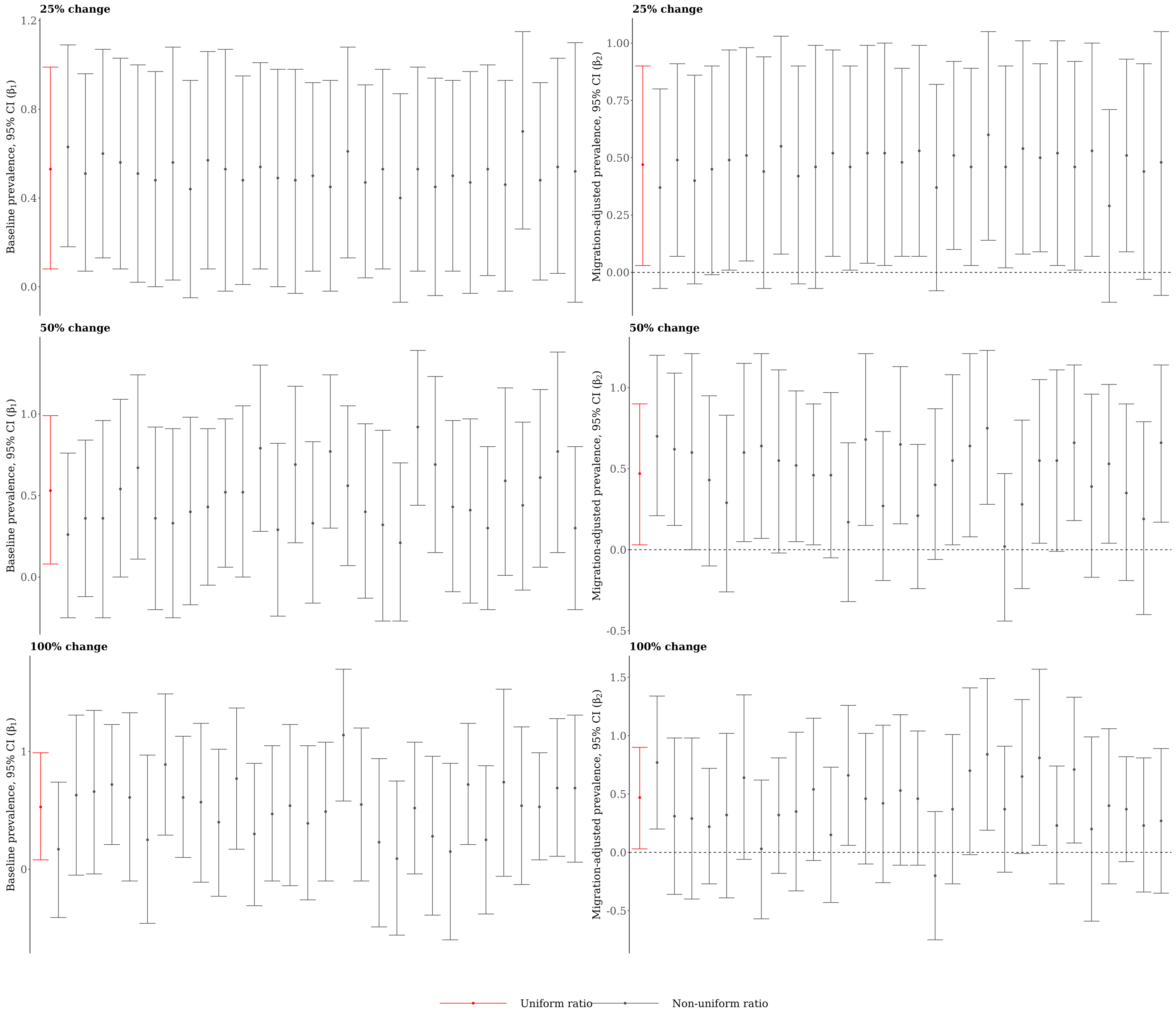


**Figure S10:** Estimated regression parameters and 95% CI for our linear regression model (equation S12, DS-TB 2016) with White-Huber robust standard errors (heteroskedasticity-consistent standard errors) where prevalence:notification ratio differs by age, sex, and municipality (see Figure S8 caption for details).

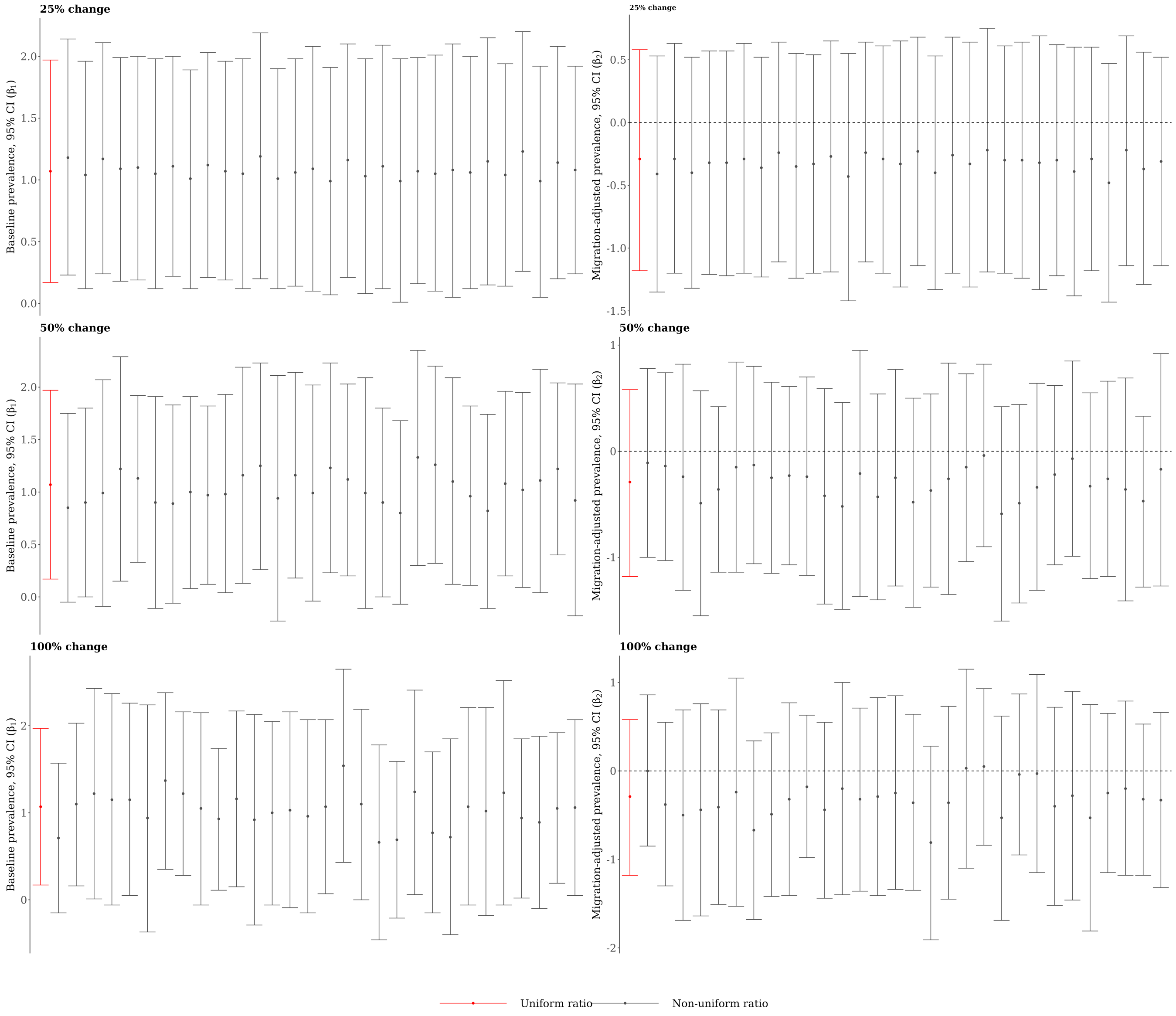


**Figure S11:** Estimated regression parameters and 95% CI for our linear regression model (equation S11, RR-TB in 2015) with White-Huber robust standard errors (heteroskedasticity-consistent standard errors) where prevalence:notification ratio differs by age, sex, and municipality (see Figure S8 caption for details).

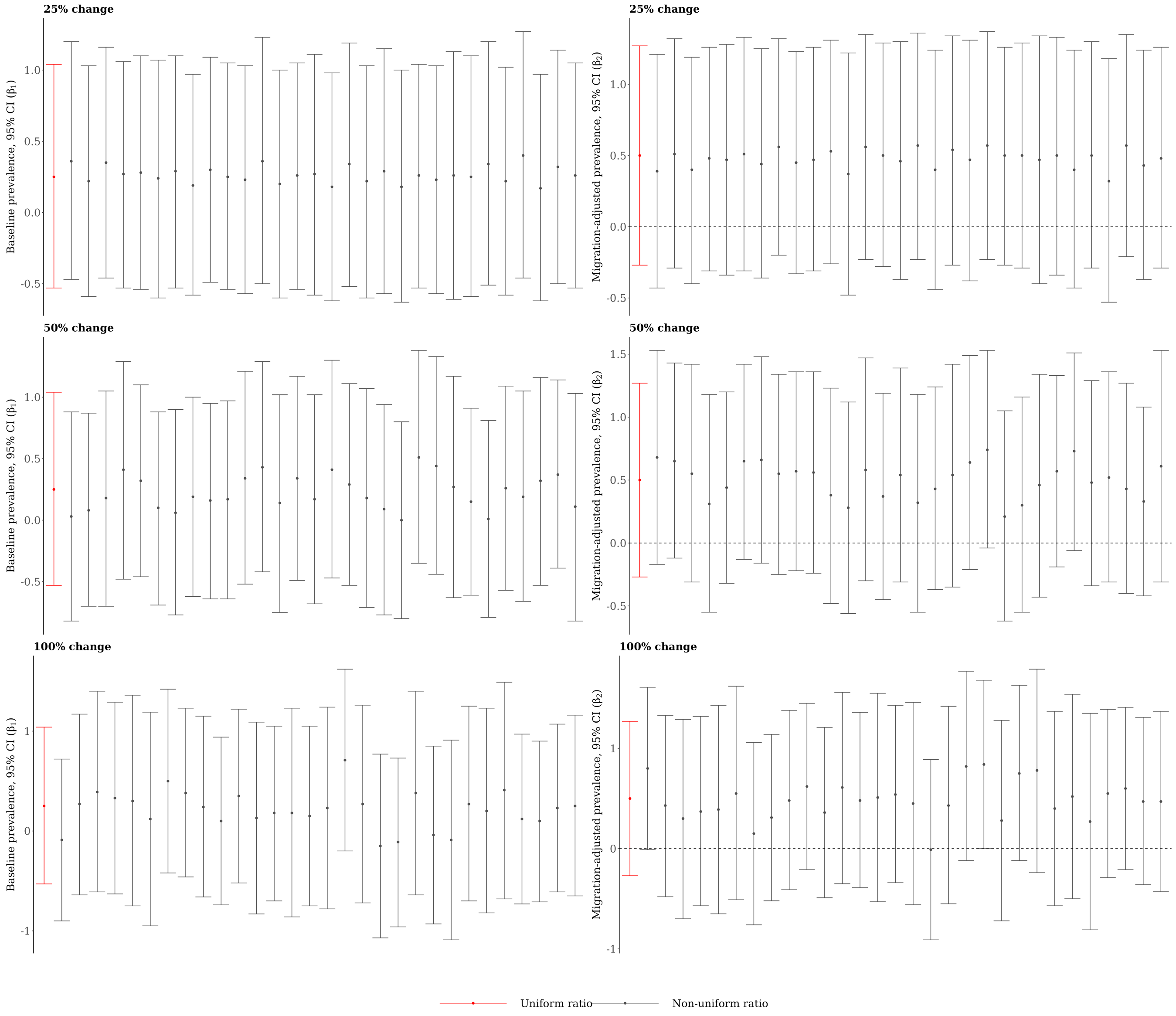


**Figure S12:** Estimated regression parameters and 95% CI for our linear regression model (equation S12, RR-TB in 2016) with White-Huber robust standard errors (heteroskedasticity-consistent standard errors) where prevalence:notification ratio differs by age, sex, and municipality (see figure S8 caption for details).

## S6. Correlation between migration rates from the HNLS and survey data

We investigated whether migration patterns in the NHLS migration cohort and household survey data (which were matched on the age, sex, and origin municipality to NHLS migration cohort) are similar. We produced binned scatter plots showing the relationship between migration rates in the NHLS migration cohort and household survey data. To do that, we grouped cross-municipality migration rates from the survey data by percentiles (100 bins). We found all the cross-municipality migration flows within each percentile from the survey data in the NHLS data, then we calculated mean migration rate for each percentile in the NHLS data. We graphed the relationship between mean migration rates in the NHLS data (y-axis) and percentiles of migration rate from the survey data (x-axis). A non-significant relationship suggests that cross-municipality migration patterns differ in the NHLS and survey data. We found that general migration patterns from the NHLS and household survey data are similar, as evidenced by a significant correlation between the two datasets (R = 0.96, see Figure S13b). Also, cross-municipality migration rates from the NHLS data are imprecise, as evidenced by higher variation in migration rates in the NHLS data (Figure S13a). This is one of the reasons why estimates of cross-municipality migration rates from matched household survey data were preferred in this analysis, as imprecise estimates introduce measurement error into our key predictor of interest (migration-adjusted prevalence).

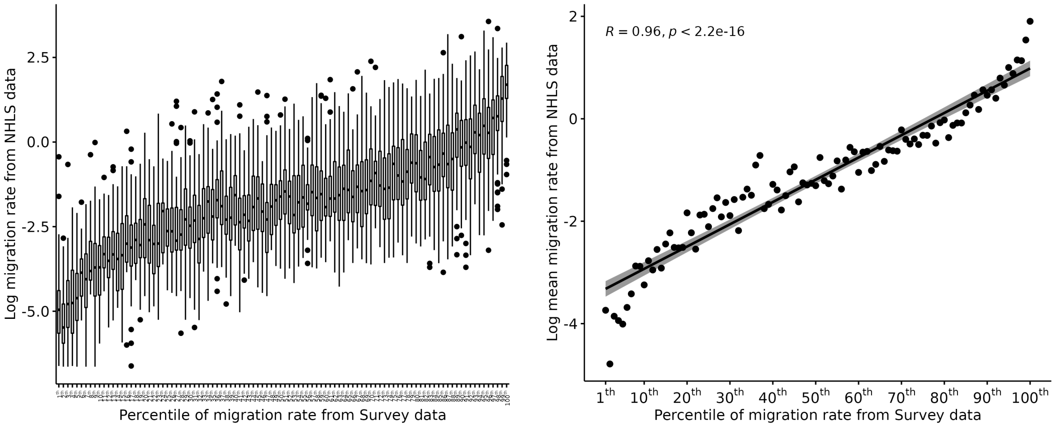


(a) (b)

**Figure S13:** Boxplots of migration rates (log) from the NHLS data by percentiles of migration rates from the survey data (a) and mean migration rates (log) from the NHLS data by percentile of migration rate from the survey data (b). In (a), lower and upper box boundaries are $25^{th}$, and $75^{th}$ percentiles of migration rates from the NHLS data. Line inside the box, lower and upper error lines are median, 10th, and 90th percentiles of migration rates respectively. Filled circles are data outside of the $10^{th}$ and $90^{th}$ percentiles. In (b), filled circle, black line, and grey area are mean migration rate, regression line and 95% confidence interval respectively. Pearson correlation coefficient (R = 0.96) quantifies the correlation between migration rates from NHLS and household survey data.

# References

[1] TB Diagnostics Market Analysis Consortium. Market assessment of tuberculosis diagnostics in South Africa, 2012-2013; 2015.

[2] Moyo S, Ismail F, Van der Walt M, Ismail N, Mkhondo N, Dlamini S, et al. Prevalence of bacteriologically confirmed pulmonary tuberculosis in South Africa, 2017–19: a multistage, cluster-based, cross-sectional survey. Lancet Infect Dis. 2022;.

[3] Harris B, Goudge J, Ataguba JE, McIntyre D, Nxumalo N, Jikwana S, et al. Inequities in access to health care in South Africa. Journal of public health policy. 2011;32(1):S102–S123.

[4] Kapwata T, Manda S. Geographic assessment of access to health care in patients with cardiovascular disease in South Africa. BMC Health Serv Res. 2018;18(1):1–10.
